# Pharmacokinetics of B-Ring Unsubstituted Flavones

**DOI:** 10.3390/pharmaceutics11080370

**Published:** 2019-08-01

**Authors:** Robert Ancuceanu, Mihaela Dinu, Cristina Dinu-Pirvu, Valentina Anuţa, Vlad Negulescu

**Affiliations:** 1 Department of Pharmaceutical Botany and Cell Biology, Faculty of Pharmacy, Carol Davila University of Medicine and Pharmacy, Bucharest, Romania; 2 Department of Physical Chemistry and Colloidal Chemistry, Faculty of Pharmacy, Carol Davila University of Medicine and Pharmacy, 020956 Bucharest 020956, Romania; 3 Department of Toxicology, Clinical Pharmacology and Psychopharmacology, Faculty of Medicine, Carol Davila University of Medicine and Pharmacy, 050474 Bucharest, Romania

**Keywords:** B-ring unsubstituted flavones, chrysin, baicalein, wogonin, oroxylin A, pharmacokinetics

## Abstract

B-ring unsubstituted flavones (of which the most widely known are chrysin, baicalein, wogonin, and oroxylin A) are 2-phenylchromen-4-one molecules of which the B-ring is devoid of any hydroxy, methoxy, or other substituent. They may be found naturally in a number of herbal products used for therapeutic purposes, and several have been designed by researchers and obtained in the laboratory. They have generated interest in the scientific community for their potential use in a variety of pathologies, and understanding their pharmacokinetics is important for a grasp of their optimal use. Based on a comprehensive survey of the relevant literature, this paper examines their absorption (with deglycosylation as a preliminary step) and their fate in the body, from metabolism to excretion. Differences among species (inter-individual) and within the same species (intra-individual) variability have been examined based on the available data, and finally, knowledge gaps and directions of future research are discussed.

## 1. Flavones as a Subgroup of Flavonoids

**Figure 1:**
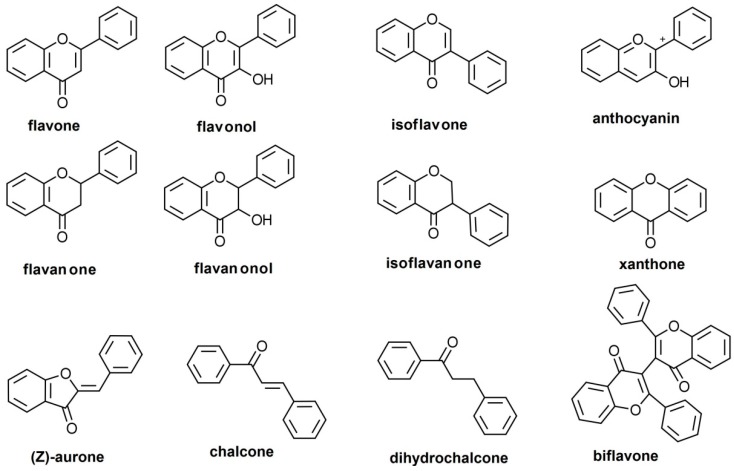
Basic chemical structures of different classes of flavonoids.

Flavonoids are a large group of natural substances derived from a 2-phenyl-chromone (flavone) nucleus, widespread in the green living world, from algae [1,2] to angiosperms [3], although the ability of algae to biosynthesize flavonoids has been controversial [4]. The basic 2-phenyl-chromone nucleus consists of a diphenylpropane (C6–C3–C6) backbone, i.e., two phenyl rings (conventionally named A and B) connected by a small chain of three carbon atoms, forming a third ring with an oxygen heteroatom (ring C) [5,6]. There is a wide range of flavonoids in nature, subdivided into multiple classes. These are defined customarily based on the position at which the B-ring connects, the cycloid character of the C-ring, and the degree of oxidation: flavones, flavonols, flavanones, flavanonols, isoflavones, isoflavanones, anthocyanidins, flavanols, xanthones, aurones, chalcones and dihydrochalcones, and biflavones (Figure 1). As a side note, although furanochromones are sometimes included among the flavonoids [6], they are not C6–C3–C6 compounds. Moreover, in a classical paper it was reported that furanochromones, although structurally close to furanocoumarins, are biosynthesized through acetate condensation and not on the phenyl-propanoid pathway [7], making questionable the inclusion of furanochromones among flavonoids.

Flavones are a group of flavonoids with the B-ring connected at the C2 position (as the majority of flavonoids have, and unlike the isoflavonoids, which have it at C3), a double bond between the C2 and C3 (unlike flavanoid derivatives), an oxidized C4 atom (like the majority of flavonoids), and no substitution at the C3 position (unlike flavonols, flavanonols, flavanols, and anthocyanidins) [8] (Figure 1). Flavones form an extensive subclass, with about 600 distinct molecules reported as of 2009 [9]. They are widespread in almost all organs and plant tissues, and they tend to occur most often as 7-O glycosides, but glycosides at any chemically conceivable position may be detected in nature, and the number of flavone glycosides exceeds the number of flavone aglycones [4]. Flavones are part of the normal human diet and are believed, based on non-clinical experiments and observational human studies, as well as a small number of clinical trials, to exert a variety of health benefits [8,10,11]. For a good understanding of the potential benefits of flavones, as well as the necessary optimal doses, it is essential that their pharmacokinetics (PK) are well understood, but their chemical diversity and large number make such an exhaustive comprehension difficult. This review will be focused on the pharmacokinetics of the subgroup of flavones with an unsubstituted B-ring.

## 2. B-Ring Unsubstituted Flavones

B-ring unsubstituted flavones are 2-phenylchromen-4-one molecules in which the B-ring does not contain any hydroxy, methoxy, or other substituent. It is assumed that the pure phenyl (with no substituents in position 2 of the flavone molecule) imprints certain chemical features which result in certain pharmacokinetic and biological characteristics, differentiating them to some extent from other flavones. For instance, the B-ring is considered the primary structural moiety that experiences biotransformation in the case of flavonoids, and the absence of hydroxyl groups on the B-cycle precludes ring scission [12].

**Figure 2:**
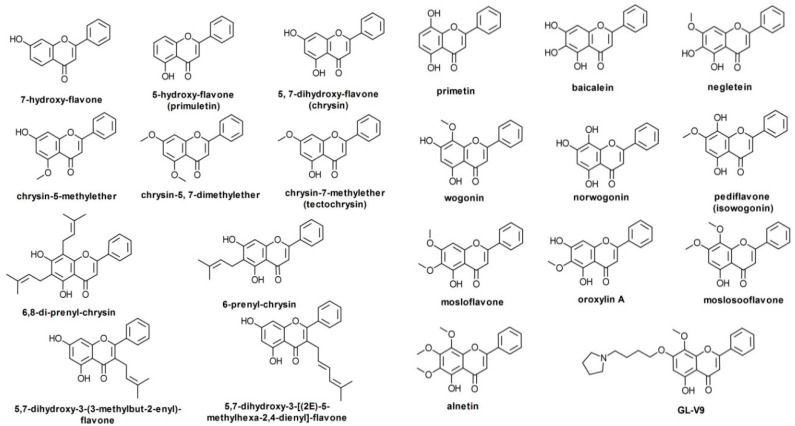
Representative chemical structures of B-ring unsubstituted flavones.

The most important such flavone aglycones, and the most widely studied up to now, are chrysin, baicalein, wogonin, and oroxylin A. Their number is larger, though, and using preliminary searches in PubMed, PubChem, Google Scholar, and a reference monograph [13], we collected over 20 such compounds (Figure 2). Chrysin has been reported as an important component of propolis and honey, as well as of herbal extracts obtained from species of genus *Passiflora* [14] or *Alpinia oxyphylla* Miquel [15]. Baicalein has been identified in several plant species to date, including *Scutellaria baicalensis* Georgi, *Scutellaria lateriflora* L., *Scutellaria barbata* D. Don, *Oroxylum indicum* (L.) Kurz, and the mushroom *Tuber aestivum* Vittad. (summer truffle) [16]. Wogonin may be extracted from species of *Scutellaria*, *Andrographis*, *Anodendron* [17], *Evodia* [18], or *Carya* [19] genera. As baicalein and wogonin, oroxylin A is also an important component of *Scutellaria* species, but is also present in species of *Oroxylum*, *Capparis*, *Ardisia*, *Stachys*, and *Eucommia* genera [20]. GL-V9 is a synthetic derivative of wogonin, with antitumor activity shown in various in vitro models [21], whereas BA-j is a synthetic derivative of baicalin (8-hydroxypiperidinemethyl-baicalein) with similar activity [22].

## 3. Search Strategy

We used each member of this collection as a keyword in PubMed, Scopus, and ISI Web of Knowledge to identify relevant publications for the absorption, distribution, metabolism, and elimination of these molecules. We then critically reviewed and summarized the data, and identified the knowledge gaps in the published literature. Understanding the pharmacokinetics of such natural compounds is important, because, as for all drugs, there may be important correlations between the PK parameters (particularly the exposure as measured by AUC) and pharmacodynamicm characteristics of herbal extracts [23].

## 4. Deglycosilation

It is generally accepted that flavonoid glycosides are subject to a deglycosylation step in order to be absorbed in the human digestive tract [24]. Pharmacokinetic studies measuring both flavone aglycones and glycosides have reported high levels of flavone glycosides (baicalin, wogonoside) in plasma [25]; however, the glycosides in these cases were actually glucuronides, and they are not absorbed as such, but rather formed in the body following absorption, and it would be naïve to imagine that they are the result of direct absorption. This makes the interpretation of PK data of herbal extracts containing both glycosides and aglycones difficult, because the glycosides measured in the blood do not directly reflect the glycosides from the extract, but they may well originate from the aglycones.

The hydrolyzing enzymes involved in this deglycosylation seem to be primarily of human origin (intestinal epithelial cells), but some reactions are catalyzed by enzymes of bacterial origin (the intestinal microbiota) [24,26]. This step is mainly catalyzed by two beta-glucosidases: the lactase-phlorizin hydrolase (LPH) and cytosolic beta-glucosidase (CBG). If the latter is located in the cytosol, the former is a membrane enzyme, located in the apical membrane of the epithelial cells of the gut [24]. Although small amounts of baicalin can be absorbed by active transport from the gut, the comparative experiments in germ-free and conventional rats have indicated that most baicalin administered orally is hydrolyzed by intestinal microbiota to its aglycone, baicalein, which is easily absorbed, and then reconverted to the glucuronide form (baicalin) in the body [27]. Baicalin seems to be rapidly metabolized by intestinal microbiota following oral administration (β-glucuronidase from *Lactobacillus brevis* is one of the enzymes that have been characterized [28], but most likely not the only one), and this process is considered essential for its absorption [29,30,31,32], which takes place by passive diffusion [33]. When baicalin was administered to germ-free rats, a very small amount of baicalin was detected in their plasma in comparison with rats with normal gut microflora (only 12% of the amount seen in normal rats was detected in germ-free rats) [27]. The volume of research on the relationship between the intestinal microbiota and the pharmacokinetics of baicalein is so extensive that the topic has been reviewed on its own by other authors, and reference is made to the relevant publications [34]. In the small intestine and in liver, wogonoside is fast hydrolyzed to wogonin, which is the only form of the flavone detected in these two organs [35]. Norwogonoside and oroxylin-A-glucuronide are also hydrolyzed by human intestinal flora, their levels decreasing considerably in parallel with an increase in the corresponding aglycones [36,37].

If this assumption (of deglycosilation as first step in the absorption process) based on limited experimental data is true, then it is more important to understand the pharmacokinetics of flavone aglycones, because following this step, different glycosides of the same aglycone will result in the same single molecule (the aglycone), following the same biological pathways in the human body, irrespective of its initial glycoside form.

## 5. Absorption

The water solubility of these flavone molecules, as for most of this chemical family, is limited. Although experimental data on their solubility are not generally available, computed solubilities using the ALOGPS algorithm show that it varies between a few mg per liter in the case of many aglycones to a little over 1 g per liter (Table 1).

Flavonoid aglycones generally tend to have good membrane permeability (as evidenced by Caco-2 cell experiments), whereas flavonoid glycosides have poor membrane permeability, related to their ability to form hydrogen bonds [38], and consequently, aglycones are absorbed better and faster [39]. In rats (similar observations were confirmed for mice [40]), t_max_ for baicalein, wogonin, and oroxylin A (aglycones) from herbal products has been estimated at about 10–15 min, whereas for baicalin and wogonoside, it was estimated to around 120 min [41,42], in line with older observations that the absorption of glycosides is much slower than that of aglycones [26]. In other experiments in rats, though, almost equally short t_max_ values (around 0.10 h, i.e., about 6 min) were reported for both glucuronides and aglycones following oral administration of a *Scutellariae radix* extract [32]. 

The absorption of pure chrysin (not conjugated with sulfate or glucuronic acid) seems to be done predominantly by passive diffusion [43]. Baicalein has good permeability as a consequence of its considerable lipophilicity, and can also be absorbed in the gut by passive diffusion; its metabolite baicalin—formed inside the intestinal epithelial cells, despite initial reports suggesting that it uses passive diffusion for transport [44]—is too polar [33,44,45] and has a larger size and a higher molecular weight, yet evidence has indicated multiple times that baicalin is the predominant metabolite in the blood flow following oral administration of baicalein or baicalin [33,45]. Based on in vitro experiments, it has been shown that the permeability coefficient of baicalein is lower than that of wogonin, which has a coefficient in turn lower than that of oroxylin A [46]. It seems that baicalin transport to the mesenteric blood is mediated primarily by MRP3 and MRP4, as well as the sinusoidal efflux from hepatocytes; baicalin may also be outflowed back from enterocytes to the intestinal lumen by the MRP2 (multidrug resistance associated protein 2) and BCRP (breast cancer resistance protein) [31,33,45,47,48]. Wogonoside formed in enterocytes from wogonin also seems to be excreted in the intestinal tract by MRP2 [35]. In MRP2-deficient rats, the AUC and C_max_ of baicalin were five-fold and eight-fold higher, respectively, than in MRP2-competent rats [47]. 

What part of the gastro-intestinal tract is involved in the absorption? In situ experiments performed in rats have indicated that baicalin is absorbed to a moderate extent in the stomach, but the absorption is weak from the small intestine and colon, whereas baicalein has good absorption from the stomach and small intestine, but its absorption is weaker from the colon [49]. In the case of baicalin (administered directly or formed from baicalein), two sites of absorption have been proposed based on the experimental data available: the upper part of the gastro-intestinal tract (including the stomach) for its direct absorption (by active transport, as discussed above), and the colon for indirect absorption (under the form of its aglycone, baicalein) [50]. The good absorption of baicalin from the stomach is interesting, because in a Caco-2 cell model it was found that its absorption was considerably better at a weakly basic pH (7.4) than at a more acidic pH (6.8 or 5.0) [44]. In mice, it has been shown that the C_max_ of baicalein is highest in the stomach, followed by jejunum, ileum, and cecum, and is lowest in the duodenum and colon [16]. GL-V9, a synthetic, non-substituted B-ring flavone investigated for its potential anticancer activities, is absorbed predominantly at the level of the duodenum and jejunum [51].

Besides the oral route, a body of research has been carried out with respect to the absorption of flavonoids by other routes. In vitro tests performed on hairless mouse skin showed that wogonin penetrated better than baicalein in vitro. In vivo, only baicalein has been evaluated, but the in vivo observations were consistent with those seen in vitro [52]. The transdermal administration of baicalin has been reported successfully in rats using a cubic phase gel [53]. Topical application in cows did not result in detectable plasma concentrations of baicalin, but it crossed the blood–milk barrier; it is absorbed slowly, and one may expect to be able to exert therapeutic effects at the place of administration [54]. The intra-tracheal administration of a traditional Chinese medicine of a herbal nature (Tanreqing solution) has shown that baicalin and oroxylin A-7-*O*-β-d-glucoronide are well and quickly absorbed into venous circulation, with no first passage effect, achieving similar exposure to i.v. administration [55]. The pulmonary route has also been explored for baicalein administered as nanocrystals [56] or (nano)liposomes [57,58], with results close to those from the i.v. route [56] for baicalin (as part of a complex herbal product) [59] and for the synthetic flavone GL-V9, where pulmonary administration almost doubled the bioavailability in comparison with the oral pathway [60]. Studies on rabbits have shown that baicalein has difficulties in penetrating the cornea when applied as a suspension, but high concentrations of the flavone in the aqueous humor and cornea could be achieved with the help of hydroxypropyl beta-cyclodextrin [61]. The use of transferosomes also may ensure fast onset of action for baicalin administered by ocular route, whereas liposomes benefitted absorption to the largest extent, with a 400–500% increase in bioavailability as compared with a baicalin control solution [62]; similar results were also reported with solid lipid nanoparticles incorporating baicalin for ocular drug delivery [63] or with the nonionic surfactant Labrasol [64].

## 6. Albumin Binding

Among several flavones of this group flavones (flavone, 7-HF, chrysin, and baicalein) 7-HF has the highest affinity for bovine and human serum albumin (BSA and HSA, respectively). For BSA, 7-HF has an affinity about 760 times higher than flavone, whereas for HSA, the affinity of 7-HF is about 43 times higher than that of flavone. Chrysin and baicalein have higher affinities for BSA and HSA than flavone, but lower than the affinities of 7-HA [65]. Computational models have suggested that chrysin binds to the IB subdomain of the albumin [66], whereas experimental data indicate that baicalein [67], wogonin [68,69], and 3,7-dihydroxyflavone [70] bind to the IIA subdomain (site I of HSA and BSA).

The affinity of (pure) chrysin for HSA is considered high (10^5^–10^6^ L/mol), but, as discussed elsewhere in this paper, the amount of chrysin as such in the blood flow is very limited, and of much more interest are its sulfate and glucuronic acid conjugates. Recent in vitro data have shown that the affinity of the chrysin sulfate conjugate (which is the primary metabolite detected in a clinical study performed in humans) for HSA is even higher than that of the parent compound [71]. Baicalein has a higher affinity for HSA binding than its glucuronic derivative, baicalin [72]. Baicalin is bound to albumin in human plasma at a proportion of about 86–92% [73], and wogonin is bound in rat plasma in proportions of about 90–94% [74].

In vitro data on a small number of flavonoid molecules have shown that glycosylation tends to decrease the affinity for BSA by one to three orders of magnitude in comparison with the aglycon (the effect depends on both the position of linking the sugar and the nature of the sugar moiety) [75].

## 7. Recycling

The half-life of flavonoids in general (as conjugates) in the body is longer than expected considering their metabolic fate, primarily because they are involved in extensive local, enteric, or enterohepatic recycling, [76,77].

**Figure 3:**
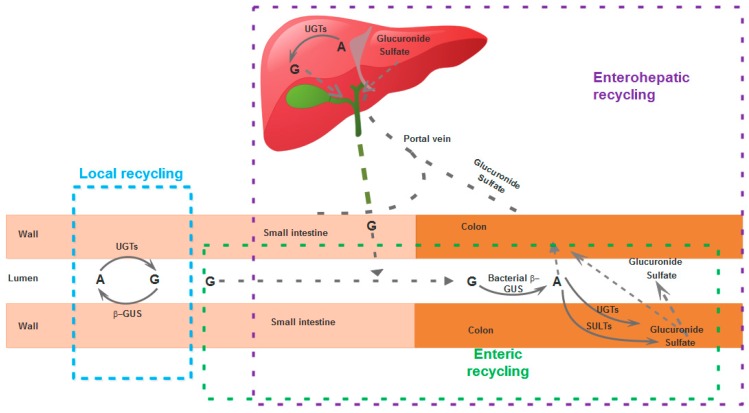
Schematic representation of different recycling scheme applicable to flavonoids. A: aglycones; G: glucuronide; β-GUS: β-glucuronidase; UGT: UDP-glucuronosyltransferases; ST: sulfotransferases.

A local recycling scheme has been proposed specifically for an unsubstituted B-ring flavone (wogonin) and its glucuronide derivative (wogonoside) based on data obtained in rats. Wogonoside is hydrolyzed quickly to wogonin, a reaction catalyzed by enterocyte-derived β-glucuronidase (β-GUS), not a bacterial-derived enzyme [76]. In enteric recycling, the flavone glucuronides are hydrolyzed back to aglycones, which then are rapidly absorbed or are re-converted back to conjugates (glucuronides, sulfates) and either effluxed to the intestinal lumen or absorbed into the blood via the colon [78]. A part of the phase II metabolites (conjugates) reaching the liver via the portal vein enter enterohepatic recycling: they are excreted via the bile into the gut, where they may be further recycled by the same pathways [79,80,81] (Figure 3). Although it has been traditionally held that glucuronides excreted in the gut through the bile are chiefly of hepatic origin, because hepatocytes generally do not take up significant amounts of glucuronides of exogenous substances, it has been demonstrated for several flavonoids, including chrysin, that glucuronides recycled enterohepatically are of extrahepatic origin [82]. The bile not only excretes baicalin and contribute to its enterohepatic recycling, but it has been shown that it promotes the absorption of baicalein [49].

These phenomena of local, enteric, or enterohepatic recycling have consequences on the pharmacokinetics of these flavones. On the one hand, they contribute to the maintenance of the compounds in the body, ensuring minimal concentrations for longer periods, but on the other hand, they complicate the pharmacokinetics, as is the case for baicalin, where multiple peaks, multiphasic decrease of plasma concentrations, and PK non-linearity have been reported [83]. Baicalin administered orally is hydrolyzed to baicalein by bacterial enzymes, but baicalein is in its turn glucuronidated to baicalin by UGTs (UDP glucuronosyltransferases) [84]. In the case of GL-V9, although a concentration peak was seen after 0.5 h of oral administration, a second twin peak was recorded after 12 h (in rats), most likely related to the enterohepatic, enteric, or local recycling [60]. A double peak was detected in the plasma concentration–time curves for baicalin, oroxin A (baicalein-7-*O*-glucoside), baicalein, wogonoside, wogonin, and chrysin when administered in rats, related to enterohepatic, local, or enteric recycling [16,23,32,85,86,87,88,89,90,91,92,93,94,95,96,97,98,99,100]. In one study in rats, it was claimed that this bi-modal “behavior” was not confirmed experimentally [101], but the authors only measured plasma samples up to 180 min, whereas in order to observe the second peak (at its maximum) at least 6–8 h would often be needed [102,103,104,105], although in other cases a second peak was reported after only two to three hours [106,107,108,109]; in dogs, following i.v. administration, a peak was seen before two hours, and a second one around four hours [110]. When wogonin was administered in beagles (intragastric route), a single peak was recorded for the free wogonin, but a double peak was observed for its main metabolite, wogonin7-β-d-glucuronic acid [111]. In rats, a double peak was reported for wogonin when the animals were administered a multi-herb traditional Chinese medicine (Tang-Min-Ling-Pill) [112]. Tectochrysin seems also to manifest enterohepatic recycling [15].

For the bimodal distribution of baicalein/baicalin, it has also been advanced that the high absorption rate of the glucuronidated form in two intestinal sites, duodenum and cecum, might explain the phenomenon [50,113]. It was acknowledged, though, that the phenomenon was seen even when the drug was administered by intravenous route and that enterohepatic recycling played a central role, because ligating the biliary tract led to the disappearance of the bimodal concentration–time curve of baicalin [113].

Following administration of a mixture of *Lonicerae flos* and *Scutellariae radix*, oroxylin-A-7-*O*-glucuronide could be detected up to 36 h post-administration, unlike baicalin and wogonoside, which could not be detected beyond 24 h post-administration [114]. In this study, oroxylin-A-7-*O*-glucuronide had not a bi-modal distribution, but a multimodal one, also attributed to various mechanisms of recycling or double absorption [114]. On the other hand, in a study of a more complex herbal product (An Gong Niu Huang Wan), baicalin was detected even after 48 h post-administration, whereas other analytes could not be detected [115].

## 8. Distribution

Three related B-ring non-substituted flavones (baicalein, wogonin and oroxylin A) have similar distributions in the body, with higher concentrations in liver and kidney than in plasma, similar concentrations in prostate and plasma, and lower concentrations in pancreas, lung, or pancreatic tumors (xenografts). These flavones are rapidly and widely distributed in the different tissues. An important proportion of the compounds were de-conjugated in the tissues, and the methylated aglycones tended to predominate in tissues in comparison with the non-methylated ones [74,116]. The aglycones were detected in the rat lung, kidney, and liver. The concentration of baicalein was higher in the lung than in kidney and liver, while the concentration of wogonin was higher in the liver than in lung or kidney [117,118]; in a study of a traditional Chinese medicine (TCM) product (‘JiangYaBiFeng’), the lung concentrations of both baicalein and baicalin were lower than those from kidney and liver [119].

In mouse, following the oral administration of a 10 mg/kg dose, 5,7-dimethoxyflavone (5,7–DMF) had a large volume of distribution (90.1 ± 62.0 L/kg), indicating extensive accumulation in the tissues [120]. It accumulates preferentially in gut, liver, spleen, kidney, brain, heart, lung, adipose tissue, plasma, and muscle (in this decreasing order) [120]. In killifish (*Fundulus heteroclitus*), a small fish used for experimental purposes, the accumulation of 5,7-DMF in organs and tissues was largely similar, unlike chrysin, which was detected in considerably lower amounts. It was found that 5,7-DMF also accumulates in important amounts in the bile, together with glucuronic acid conjugates of the *O*-demethylated derivatives (in positions 5 or 7), whereas in the case of chrysin, only the glucuronidated derivatives were detected [121].

Of the two B-ring unsubstituted flavones from *Alpinia oxyphylla*—chrysin and tectochrysin—only the latter one was shown to cross the blood–brain barrier in rat, but the levels reached there were rather low [15]. Tectochrysin is also the only one of the three reaching the fat and testis, in fairly small amounts [15].

In vitro data have shown that the three related B-ring non-substituted flavones from *S. baicalensis* (baicalein, wogonin, and oroxylin A) are able to pass the blood–brain barrier, as well as their glucuronide conjugates [74,122]. In vivo data obtained in rats [123] and rabbits [124] confirm that baicalein crosses the blood–brain barrier [123], in one study in 20–30 min following i.v. administration; the amounts reaching the brain are relatively small as compared with the blood levels (AUC brain/AUC blood = 0.023), but the brain concentration doubles when co-administered with a P-glycoprotein inhibitor (cyclosporin A) (AUC brain/AUC blood = 0.049) [125]. Baicalin accumulates in relevant amounts in certain brain areas, particularly in the striatum, thalamus, and hippocampus [126].

The results on brain distribution with herbal extracts containing the three main flavones from *S. baicalensis* were rather different from these studies performed with baicalin administered as a single substance. In one study, all the three aglycones (baicalein, wogonin, and oroxylin A) were detected in the rat brain, as well as wogonoside, but not baicalin; instead, 2′,3′-dihydroxybaicalin, a metabolite of baicalin, was detected in the brain [127]. In another study (in rat), baicalin, wogonin, wogonoside, oroxylin A, and oroxylin A-7-*O*-glucuronide were detected in the brain, in relatively small amounts compared with the plasma concentrations, indicating a low blood–brain permeability; baicalein was detected, but could not be quantified [128]. Among the three aglycones and their glucuronides, oroxylin A reached the highest concentrations in the brain tissues, despite the fact that its concentration was more limited in the herbal extract used as a source for those flavones (the brain-to-plasma ratio after 6 h of administration was about 0.45) [122]. In a PK study of *Scutellariae radix*, the three main flavones and glucuronides were detected in the brain, but wogonin and wogonoside were under the lower limit of quantification [129]. However, in a repeated dose study (seven doses) carried out in rats, no free or conjugate baicalein and wogonin were detected in the brains of the animals [117]. This might be related to the sensitivity of the method used for detection, which was clearly inferior to at least one used in a study were the flavones could be detected in the brain [129]. Using tocol nanostructured lipid carriers, a two- to three-fold increase in brain levels of baicalein was reported [130]; using nanocrystal suspensions modified with surfactants ensured an increase in the brain exposure of baicalin of about seven times higher than that of a solution [131]; using cationic solid lipid nanoparticles conjugated with an antibody, an exposure 11 times higher than that obtained with a control solution was reached in the cerebrospinal fluid [132]. The olfactory pathway has also been explored for the circumvention of the blood–brain barrier, using a phospholipid complex with the flavone molecule, with reportedly good results [133].

Following intra-gastric administration, baicalin may reach therapeutically relevant concentrations in the lens in about 1 h, as shown in rabbits [134].

In vitro data on HepG2 culture cells have shown that inside the cells, oroxylin A is distributed predominantly in the nucleus, whereas its glucuronide metabolite (oroxylin A 7-*O*-d-glucuronide) accumulates predominantly in mitochondria [135].

## 9. Metabolism and Stability to Metabolic Attack

All flavones, including those with a non-substituted B-ring, are rapidly metabolized in the small intestine (in the jejunal and ileal portions), and then are supplementarily biotransformed in the liver, whereas those not absorbed experience further metabolism under the bacterial enzymes from the colon [12]. The extended first-pass biotransformation of flavonoids, mostly by glucuronidation and sulfation, has been shown to be the primary reason for the meager bioavailability of flavonoids [136]. The synthetic flavone GL-V9 is also well absorbed at the intestinal level in rats, but extensive liver metabolism leads to its reduced systemic exposure [51].

In vitro data show that for both B-ring unsubstituted flavones and B-ring substituted flavones, the non-methylated compounds (i.e., those having only hydroxyl groups) are considerably less stable in the presence of metabolizing enzymes (such as the S9 liver fraction) than their methylated counterparts. Thus, chrysin completely disappears in about 20 min, followed by 7-HF and apigenin, whereas after 60 min of incubation, less than 20% of the corresponding methylated flavones (5,7-dimethyl-flavone, 7-methyl-flavone, and 5,7,4′-trimethyl-flavone) disappear from the reaction medium [137]. For flavonoids in general, including unsubstituted B-ring flavones, methoxylated derivatives, such as wogonin, tend to be more stable under the action of human gut microflora than their hydroxylated equivalents [138]. The microbial degradation of chrysin seems to be slower than that of apigenin, suggesting that the 4′-hydroxyl group on the B ring is consequential for rapid microbial degradation when the 5- and 7- positions are occupied by hydroxyl groups [138].

In an old publication (dating from 1972), it was claimed that following administration of chrysin to rats (200 mg by stomach tube), apigenin (i.e., hydroxylation on the B-ring in position 4′) and unchanged chrysin were detected in the urine of the animals [139]. These findings are quite different from what more modern studies have shown: whereas small amounts of unchanged chrysin are not surprising, as shown below, no modern study seems to have reported formation of apigenin by the biotransformation of chrysin.

The metabolites of baicalein (and the situation is likely to be the same for other flavones) vary in their structure and composition across the gastro-intestinal tract of mice: glucuronidated and glucosidated metabolites are dominant in the small intestine; dehydroxylated metabolites are more abundant in the large intestine; whereas methylated and sulfated metabolites are present in similar abundance in the whole intestine [16].

There seems to be an important contribution to the metabolism of flavonoid compounds from the intestinal microbiota. Wogonin (as well as diosmetin, which does not have an unsubstituted B ring) was among the most stable to rat, rabbit, and human feces suspensions, whereas 11 other flavones of different structures (including baicaline) were extensively degraded by the microbiota from these feces [140]. A methoxyl group on the A or B rings (as is the case for both wogonin and diosmetin) seems to confer protection to microbial degradation. Although hesperetin also has a methoxy group, it was more stable in rabbit feces, but degraded in rat and human feces [140].

Rat perfusion experimental data indicated that a higher loading dose of baicalein leads to diminished first-pass metabolism in intestine, suggesting a saturation effect with high doses [141].

An in vitro study with rat microsomes indicated that baicalin may be influenced by CYP3A4, CYP1A2, CYP2C19, and UGT1A1, UGT1A3, and UGT1A9; baicalein, instead, is metabolized under the influence of CYP3A4, CYP2E1, and UGT1A6 [142]. Baicalin induces the expression of CYP2B6 and inhibits the expression of CYP2E1, which opens the door for various herb–herb or herb–drug interactions, where baicalin exerts an influence on other herbal ingredients or drugs [143,144]; these aspects are outside of the scope of this paper.

### 9.1. Glucuronidation

Glucuronidation of flavones by UDP-glucuronosyltransferases (UGTs) represents (together with sulfation) the most important metabolic pathway of these natural molecules when ingested by humans [145,146], because phase II metabolism reactions are more important for these natural substances than phase I reactions [147]. In the case of the main *Scutellariae radix* flavones (baicalein, wogonin, and oroxylin A, and their glycosides baicalin, wogonoside, and oroxylin A 7-*O*-β-d-glucuronide), following oral administration of an extract to rats, the C_max_ and AUC0-48h of the glucuronides were at least 10 times higher (and up to 130 times higher) than those of the respective aglycones [32]. The glucuronidation of wogonin to its phase II conjugate, wogonoside, was shown in rats to take place very fast [35]. The highest clearance by formation of glucuronides was for oroxylin A, about 10 times higher than that of baicalein [46]. Chrysin and tectochrysin are also conjugated with glucuronic acid in rats [148].

Drug disposal in general, and flavone disposal in particular, via glucuronidation involves two steps: the formation of glucuronide conjugates (discussed above) and the excretion of the glucuronide conjugates out of the cell via efflux transporters (passive transport is not a viable option for glucuronides, because of their high hydrophylicity) [149].

Of the four UGT families (UGT1, UGT2, UGT3, and UGT8), the UGT1A subfamily (but excluding UGT1A4, and UGT1A6) is mostly involved in the glucuronidation of flavonoids [150]. UGT1A1, UGT1A7, UGT1A8, UGT1A9, UGT1A10, and UGT2B7 seem to be the crucial isoforms involved in glucuronidation of hydroxyflavones [145]; UGT1A3 and UGT1A7-1A10 were shown by in vitro experimental data to be the main families involved in the glucuronidation of wogonin and oroxylin A [136]. Glucuronidation of baicalein to baicalin is catalyzed primarily by UGT 1A9, and secondarily by UGT 1A1, 1A3, 1A8, 1A7, and 2B15 [45].

Rat data (not obtained with unsubstituted B-ring flavones) suggest that intestinal UGT enzymes are more important than their liver counterparts, and that animals deficient in UGT1A isoforms overexpress UGT2B, which may be equally effective in the glucuronidation of flavones [151]. However, it has been shown that in vitro glucuronidation (as well as sulfation) takes place much faster in hepatic microsomes than in the gut microsomes [45,152]. 3,7-dihydroxyflavone (resogalangin) and 5,7-dihydroxyflavone (chrysin) are predominantly glucuronidated by UGT1A1 [150], but in Caco-2 cells, where UGT1A6 is the only UGT isoform expressed, this isoform was responsible for the glucuronidation of chrysin [153]; as discussed below, human clinical data on a small number of volunteers have demonstrated that chrysin is mostly sulfated and the contribution of the glucuronide pathway to its metabolism is negligible [154].

Not only is chrysin glucuronidated in vitro by UGT1A1, but it seems to be an inductor of this UGT isoform, as shown by experiments carried out in Caco-2 and Hep G2 cells. This inductor effect of chrysin is not related to its unsubstituted B-ring though, but rather to its 5,7-dihydroxy-flavone backbone; it has been shown that among a variety of flavones (*n* = 22), only four, all with this 5,7-dihydroxy-flavone structure, were able to induce three- to five-fold the glucuronidation in intact cells. A partially methylated derivative (5-hydroxy-7-methoxy-flavone) had a more modest effect (1.5–2 increase in glucuronidation), whereas all other flavonoids tested had no inductor effect [155].

Enzymes involved in glucuronidation of flavonoids express particular regioselectivities, with UGT1A3 and UGT1A9 favoring the 7-OH-glucuronidation, while UGT1A7 prefers the 3-OH group; because flavones do not have a hydroxyl group at C3, the latter enzyme group (UGT1A7) is of little relevance for flavones [156,157,158]. UGT1A9 is more efficient in glucuronidation than UGT1A3 [158]. UGT1A8 and UGT1A9 also seem to have a weak or moderate preference for the 3-O position versus the 7-O position, whereas UGT1A1 and other isoforms seem to lack a preference for certain specific positions. However, as a general rule, glucuronidating enzymes seem to prefer the 7-O, 3-O, and 4′-O positions, whereas the 5-O is disfavored [156,157,159]; because in the case of unsubstituted B-ring flavones, no 3-O and 4′-O positions are available, the 7-O position becomes the preferred site of glucuronidation for this group of flavones, but forming small amounts of 5-*O*-glucuronides is possible and has been reported [160]. Baicalin, the mono-glucuronide of baicalein, which has the 7-O position already glucuronidated, was reported to bear no conjugation under the influence of UGT1A3 and UGT1A9 [161]; this is consistent with the preference of UGTs for hydroxylating the 7-O position. However, the position of glucuronidation in the case of baicalein is also dependent on the different UGT isoforms: UGT1A1, UGT1A3, and UGT1A6 catalyze glucuronidation exclusively at the 7-O position; UGT1A8, UGT1A9, and UGT1A10 catalyze monoglucuronidation at positions 7-O or 6-O. UGT1A8, though, has a preference for the 6-*O*-position (70% of the monoglucuronides formed), whereas UGT1A9 and UGT1A10 have a marked preference for the 7-O position (95% and 80% of the monoglucuronides formed) [159]. The synthetic flavone GL-V9 has a free hydroxyl available only in position 5, and although this is not a preferred position of glucuronidation, almost 90% of this flavone is glucuronidated in that position, as shown when administered in rat [60]. Unlike GL-V9, another synthetic derivative, 8-hydroxypiperidinylmethyl-baicalein (BA-j), although has three free hydroxyl groups, it is glucuronidated only in proportions of less than 2–3% [162].

As a consequence of these regioselectivities in glucuronidation of flavonoids, UGT1A8 and UGT1A9 seem to be the dominant isoforms involved in the glucuronidation of wogonin and oroxylin A, whereas UGT1A1 and UGT1A9 are involved in the glucuronidation of baicalein [163].

An in vitro study investigating a number of seven mono- and di-hydroxy-flavones has found that in the case of both mono-hydroxylated derivatives, as in the one of the bi-hydroxylated compounds, only mono-glucuronides were formed. Although no di-glucuronide seems to be biosynthesized, a second, minor glucuronide was detected besides the primary, major one for the dihydroxylated flavone molecules. At all concentrations evaluated, the 3-HF (the prototype molecule of flavonols) was metabolized in the shortest time by all UGT enzymes assessed, while the 5-HF (as well as 4′-HF, which has a hydroxyl group on the B-ring) was metabolized in a slower manner by the UGT isoforms; in other words, flavonols tend to be glucuronidated faster than flavones [145]. Glucuronidation by liver microsome enzymes was also fastest for the 3-HF and slowest for the 5-HF. The authors speculated that the reduced metabolization of the 5-hydroxy derivatives was related to the formation of a hydrogen-bond within the same molecule between the C-5 hydroxyl group and the C-4 carbonyl group; the C-4 carbonyl group may alternatively or cumulatively represent a steric impediment for the access of the glucuronidation enzyme [145]. In any case, the low reactivity of the C-5 hydroxyl group of flavones is well-known, chemical derivatization in this position through acetylation or methylation being notoriously difficult [164].

An in vitro study performed with human jejunum S9 fraction, showed that chrysin (5,7-DHF) is glucuronidated slightly slower than 3′,7-DHF (hydroxylated on the B-ring in the 3′ position), but faster than 2′,7-DHF, 6,7-DHF, 7,8-DHF, 7-HF, 4′,7-DHF, and 3,7-DHF (written in decreasing order of biotransformation) [160]. A Caco-2 cell model comparing the glucuronidation of chrysin, luteolin, apigenin, and baicalein found that among the four flavones tested, chrysin had the higher total amount of glucuronides, whereas baicalin had the lowest amount [165].

Following administration of baicalin or baicalein in rats, baicalein 6-*O*-β-d–glucopyranuronoside was detected as a main metabolite, in higher amounts than baicalin, and its origin was mostly hepatic and not intestinal [32,166]. In the same species, wogonoside (oroxindin) has been reported as a main metabolite of baicalein, in concentrations close to those of baicalin [167]. In the intestine of mouse, the most important metabolites detected were baicalein-6,7-di-*O*-glucuronide, followed by baicalein-glucuronide-methylate, baicalin, and oroxin A; in smaller amounts, baicalein-glucuronide-glucoside, baicalein-sulfate, chrysin, baicalein-6-*O*-glucuronide, oroxylin A, and luteolin [16].

In humans, following the administration of baicalein (or baicalin), five main metabolites have been detected in plasma, with baicalein as the most important. The others are baicalein-6,7-di-*O*-glucuronide, baicalein-6-*O*-glucuronide, 6-methoxybaicalein-7-*O*-glucuronide, and 7-methoxybaicalein 6-*O*-glucuronide [81,168]. The authors proposed that baicalein is first mono-glucuronidated in positions 6 or 7 (forming baicalin or baicalein-6-*O*-glucuronide, respectively), and then the monoglucuronide derivatives are methoxylated in position 6 or 7, or a second glucuronic moiety is added (also in position 6 or 7) [168]. Most of these five main metabolites have also been detected in rat bile, except for 7-methoxybaicalein 6-*O*-glucuronide; instead, 6-*O*-beta-glucopyranuronosyl- baicalein 7-*O*-sulfate was additionally reported in the bile [169].

A rat study using a UHPLC-MS method identified no fewer than 32 metabolites of baicalin, of which five were detected in the heart, nine in the liver, five in the spleen, five in the lung, five in the brain, and ten in the kidney. Most of them were glucuronide conjugates, although sulfate conjugates and non-conjugate metabolites were also detected. The authors assumed that six of them (5-hydroxyflavone-6-*O*-glucoside-7-*O*-glucuronide; 5-hydroxyflavone-6-*O*-glucopyranuronoside-7-*O*-glucopyranuronoside; 5,6-dihydroxyflavone 7-*O*-glucoside; 5-hydroxy-6-methoxyflavone-7-*O*-glucuronide; 5,6-dihydroxyflavone-7-*O*-glucuronide; and 5,7-dihydroxy-6-methoxyflavone) were relevant for the pharmacological activity of baicalin, an assumption based on the wider distribution in the different tissues of these metabolites [170]. Wogonin and oroxylin A, structurally related flavones to baicalein, are also (based on in situ rat data) glucuronidated, and glucuronic conjugates are found in the mesenteric blood in remarkably higher amounts than the parent compounds [46]. Data from beagles indicate that most wogonin administered by intragastric route is rapidly converted in vivo to the 7-*O*-glucuronide derivative [111], but smaller amounts of the 5-*O*-glucuronide derivatives seem also to be found (rat data); instead, oroxylin A seems to be conjugated with glucuronic acid only in the 7-*O*- position [171,172]. In the rat intestine, glucuronides and their aglycones may be converted to each other; at least, this was demonstrated for wogonin and its 7-*O*-glucuronide (wogonoside) [76], and this may be part of an enterohepatic recycling.

Saturation of the glucuronidation reactions have been detected in vitro (with human jejunum S9 fraction) for chrysin and 7-HF (as well as for flavones substituted on the B ring), but no saturation was observed for 6,7-DHF and 7,8-DHF between concentrations of 2 and 59 μM [160]. The somewhat equivocal results for three different doses of baicalein (200, 400, and 800 mg) administered to healthy subjects have been interpreted in the literature as not supporting a saturation of the conversion reaction of baicalein to baicalin [173].

### 9.2. Sulfation

Sulfation is a second contributor to flavone metabolism, in addition to glucuronidation, which has generally tended to be more prominent in non-clinical studies. Sulfation reactions are catalyzed by cytosolic sulfotransferases (SULTs), of which 14 different enzymes are known in humans to date, organized into four families: SULT1, SULT2, SULT4, and SULT6 [174]. The first two families are richly expressed in human hepatocytes and intestinal cells, whereas the role(s) and characteristics of the latter ones are currently less understood [174]. The SULT1 family is, in its turn, subdivided into eight subfamilies: A1–A4, B1, C2, C4, and E1 [175].

Clinical data obtained in humans with an oral dose of 400 mg of chrysin showed that the major metabolism pathway of this unsubstituted B-ring flavone is sulfation and not glucuronidation. The parent compound may be present in low amounts in plasma, but the concentration of sulfate conjugates was about 30 times higher than that of the parent compound (between 420 and 4220 ng·mL^−1^·h for the sulfate derivative, and only between 3 and 16 ng·mL^−1^·h for the parent compound). Instead, despite the fact that some of the patient samples did contain small amounts of glucuronide conjugates, those amounts were under the limit of quantification, and thus the role of glucuronidation in the case of chrysin is negligible [154].

In vitro data have suggested that baicalein may also be sulfated in addition to glucuronidation, although sulfate conjugates seem to be minor when compared with the glucuronide conjugates [46,152,176]. Additionally, Caco-2 cell data have suggested that baicalein–sulfate tends to follow the apical efflux (towards the intestinal lumen), whereas baicalin tends to follow the baso-lateral efflux (towards the mesenteric blood) [45]. Similarly to the chrysin case, human PK data (*n* = 10 volunteers) based on administration of *Scutellariae radix* also indicated that, contrary to non-clinical data, the sulfate conjugates are dominant in comparison with the glucuronides, although the glucuronides are present in important amounts, unlike chrysin (of which the amount was negligible in humans) [177]. A repeated dose PK study of *Scutellariae radix* extract (seven doses) performed in rats also found that all important organs contained more sulfates than glucuronides [117].

For the synthetic flavone GL-V9, no sulfated or methylated phase II metabolite was detected when administered in rats, suggesting that these metabolic pathways are unlikely to be relevant for this substance [60]. It is not possible to assess whether the same is true in humans.

PK studies performed in rats with 5-HF and 7-HF have shown that neither of the two parent compounds are detected in serum, only the conjugated forms: glucuronides for 5-HF and both glucuronides and sulfate conjugates in the case of 7-HF (in the case of 5-HF, the sulfate contribution was minimal) [164]. The metabolism of the two flavones (5-HF and 7-HF) is similar in humans: only the 7-HF is sulfated, in addition to glucuronidation, whereas the 5-HF is only conjugated with glucuronic acid. Flavonols (which are flavones hydroxylated in position 3) are also only glucuronidated, but not sulfated; contrarily, flavonols 3-HF and 5-HF are inhibitors of the sulfation enzyme SULTA1 [178]. Both SULTA1 and SULTE1 have been shown to sulfate various flavonoids, including chrysin, with SULTA1 having greater activity than SULTE1 [179].

In Caco-2 cells, it was observed that wogonin and oroxylin A are also sulfated, besides glucuronidation; however, sulfate conjugates were not detected in a rat intestinal perfusion model [46].

### 9.3. Demethylation

It has been reported, based on in vitro data (with human liver S9 fractions), that fully methylated flavones are largely stable to biotransformation, unlike hydroxylated flavones (including partially methylated ones). Moreover, 5,7-DMF, an unsubstituted B-ring flavone, was considerably more stable than 3′,4′-DMF (a flavone substituted on the B-ring) [180]. In a study evaluating 15 different methoxylated flavones, 5,7-DMF and 5-MF were shown to be the most stable to biotransformation by hepatic microsomes, 81% and 74% of their initial amounts persisting unmodified after incubation for 30 min with the microsomes. In this study, 7-MF had a much shorter half-life than 5-MF (was much more unstable), whereas tectochrysin, which, besides a methoxy group in position 7, also has a hydroxyl in position 5, had the shortest half-life (was the most unstable). It is not clear why kaempferide, with three hydroxyl groups, was more stable metabolically than tectochrysin [181]. 5,7-DMF is metabolized in vitro by CYP450 isoforms, the most important fraction being CYP1A1, with smaller contributions of CYP1A2 and CYP3A4. 5,7-DMF is primarily metabolized by these CYP450 isoforms by 7-*O*-demethylation to 5-methoxy-7-hydroxyflavone, which then seems to be further metabolized to more polar compounds [181].

However, it is not clear to what extent these findings are relevant when such methylated flavones are administered to humans, because it has been shown that total or partially methoxylated flavones, such as 5,7-dimethoxy flavone (5,7-DMF) or 5-hydroxy-7-methoxy flavone (5-OH-7-MF, tectochrysin), are demethylated (*O*-methyl hydrolysis) to chrysin under the influence of the strict anaerobic intestinal bacteria of the *Blautia sp.* [182]. The complete demethylation required about two days. 3,5,7-trimethoxy-flavone and 5-OH-3,7-DMF are also sequentially demethylated under the action of intestinal *Blautia* sp., with a region selectivity decreasing in the following order: C-7, C-5, and C-3 [183]. It is difficult for the moment to assess the full clinical significance of such findings relating to demethylation. First, these observations come from in vitro data, and such data most often have to be regarded with a dose of caution as to their direct in vivo relevance. Despite the fact that bacteria from *Blautia* sp. are among the most prolific components of the gastro-intestinal microbiota (representing somewhere between 2.5% and 16% of the total microbial organisms of the gastro-intestinal tract) [184], it is not clear how much of the demethylation process can take place before the methylated flavonoids are absorbed into the blood flow, because they will rarely stay very long in the small intestine (where it is assumed that most of the absorption takes place): the small intestine transit duration varies between less than one hour and more than seven hours, with a median time of about three hours [185]. Is the median duration of three hours sufficient to achieve clinically relevant demethylation (when for the complete process two days are necessary)? No such data seem to have been published as yet.

### 9.4. Methylation and Other Biotransformations

Besides conjugation with glucuronic or sulfuric acids or demethylation, baicalin is subject to methylation and methoxylation, hydrolysis, and hydroxylation [170]. In one rat study, 27 metabolites of baicalin were detected. Among them, two were methylation products, four were methylation metabolites, and an additional five were the product of methylation and hydroxylation reactions [186]. The hydroxylation of baicalin in position 4′ (to scutellarin) has been reported in rats, and it is one of the few cases where the non-polar, unsubstituted B-ring is chemically biotransformed by hydroxylation, followed by methylation to a methoxy- group [170]. The 4′- hydroxylated and methoxylated metabolites were detected in plasma, but in no other organ of the rats [170]. Three baicalin isomers were also detected among the baicalin metabolites in rats [186].

Baicalein (either administered as such or formed by the bacterial hydrolysis of baicalin) is rapidly glucuronidated in position 7 after a slow conversion (by COMT) to oroxylin A (7-methoxy-baicalein). In other words, baicalein is first methoxylated to oroxylin A, a reaction that takes place slowly, and oroxylin A is then rapidly glucuronidated to oroxylin A-7-*O*-β-d-glucuronide [29]. In vitro data generated with a microsome–hydrogel system have identified a metabolite of oroxylin A, named M318 by the authors (with a chemical structure of 2′,3′-dihidroxy-oroxylin A), which was shown to have higher cytotoxicity than its parent compound, but which was unstable and converted to an M300 metabolite (3′-hidroxy-oroxylin A) [187].

Unlike natural B-unsubstituted flavones and unlike GL-V9, BA-j is scarcely glucuronidated; its main biotransformation implies the formation of some dihydroflavonol derivatives that are then further degraded into the main metabolite, M179, by Schiff reaction and the second metabolite (of importance), M264, by sulfation [162]. In this biotransformation, several molecules of H_2_O_2_ are released, which has been suggested to be a mechanism by which this synthetic flavone may induce apoptosis and slow down tumor proliferation [162].

## 10. Excretion

General data from flavonoids and a few studies with B-ring unsubstituted flavones indicate that most of these molecule glucuronidates are subject to biliary excretion, with little urinary contribution. For instance, in the case of chrysin, only about 3% of the dose administered is excreted through kidneys [82,154].

In past, it has also been shown that baicalin is excreted primarily (about half of an administered dose in the first 30 h following administration) to the bile in the form of glucuronide conjugates (mainly 6-*O*-beta-glucopyranuronosyl-baicalein 7-*O*-sulfate and baicalein 6,7-di-*O*-beta-glucopyranuronoside) [169]. Baicalein is excreted unchanged in urine in very low amounts (0.7%), whereas about a quarter of the administered dose (27.1%) is excreted unchanged in feces [83]. When the amounts of all conjugates of baicaline detected in urine (following administration of a *S. baicalensis* extract) were summed up, they only accounted for about 7% of the content of the aglycone and glucuronide initially present in the administered extract [177]. It is assumed, therefore, that the largest part of the baicalin (from the initial administration or from the conversion of baicalein) is excreted in feces directly or following enterohepatic recycling, or is metabolized by the intestinal microbiota, which may degrade about half of a dose in 24 h, as indicated by in vitro experiments [140,188]. Intestinal microflora may degrade baicalin first to baicalein, then to 3,4-dihydroxybenzoic acid, pyrogallol, and phenylacetic acid [189].

Baicalin and wogonoside reach relatively high levels in urine, whereas their aglycones, baicalein and wogonin, are excreted through urine only in small amounts. Instead, the aglycones are found in higher amounts in feces, where the glycosides have comparatively lower levels [190]. Only about 21% of the wogonin administered i.v. (single dose, 20 mg/kg) was excreted in an unmodified form through feces, urine, and bile in rats [74].

The secretion of baicalin and wogonoside to the bile is mediated by MRPs and BCRP, as well as by OATP2B1 and OATP1B3 from liver [35,152]. Circumstantial evidence also indicates that OATP2B1 and OATP1B3 are involved in transporting wogonoside into the liver through the portal vein [35]. In HeLa cells transfected with UGT1A1, it has been demonstrated that several transporters are involved in chrysin glucuronide excretion: BCRP, MRP1, MRP3, and MRP4 [149]. Limited data suggest that the same transporters are involved in the efflux of oroxylin-A 7-*O*-beta-d-glucopyranosiduronide as those of baicalin or chrysin [38]. The cellular excretion of sulfate conjugates is mostly mediated by the multi-drug resistance protein MRP4, whereas the breast cancer resistance protein (BCRP) contribution seems negligible [174].

In humans, median t_max_ for tablets administered orally at three single doses (200, 400, and 800 mg) varied between 1.25 and 4 h for baicalein and between 1 and 4 h for baicalin. The differences when compared with rat data were not so substantial; however, the t_1/2_ was considerably longer in humans as compared with the t_1/2_ reported in most rat studies: it ranged between 8.54 and 18.85 h for baicalein and between 9.69 and 16.35 h for baicalin [191]. The PK of wogonin in rats seems to be linear, and multiple data have shown that wogonin is absorbed rapidly following oral administration (t_max_ varying between 0.22 and 0.86 h), and the half-life varies considerably (from 0.47 to 9.13 h). However, most of these studies were performed with extracts, and wogonin was not previously accurately quantified in those extracts [192]. Following its i.v. administration (20 mg/kg), wogonin is completely cleared from plasma in about 4–5 h [193]. In mouse, following the oral administration of a 10 mg/kg dose, 5,7-DMF has a very short t_max_, (0.14–0.36 h), but a half-life of 3.40 ± 2.80 h, which means that about 17 h are necessary to clear the compound quasi-completely (>95%) from the body [120].

The excretion rate varies depending on the ingredient matrix in which flavones are found: pure baicalin is excreted faster than when the flavone is part of a herbal extract (such as *Scutellariae radix*) [190].

For other flavones, we did not find direct experimental data on excretion, but it is likely that they are also primarily excreted through the bile and intestinal tract, with little contribution from the urinary pathway.

## 11. Bioavailability

Rat data derived from a small study indicated that the bioavailability of mono-hydroxylated unsubstituted B-ring flavones (5-HF and 7-HF) (as conjugates) was considerably lower than that of a tetrahydroxylated flavone on both A and B rings (fisetine). After administration of the same dose, the bioavailability of 7-HF was even lower than that of 5-HF. It has been speculated that these differences are related to the different solubilities of the three compounds, but differences in metabolism might as well explain the disparity in bioavailability [164], because whereas fisetin is indeed considerably more soluble in water than the other two, the differences in solubility between 5-HF and 7-HF are very small, and the predicted solubility of 7-HF is slightly higher than that of 5-HF (see Table 1), whereas its reported bioavailability was lower.

Following oral administration of an *Alpinia oxyphylla* fruit extract in rats, small amounts of free chrysin and tectochrysin were detected in plasma (whereas izalpinin, which is an unsubstituted B-ring flavonol, was almost undetected) [148].

Baicalein (5,6,7-trihydroxy-flavone) has also low bioavailability, attributed to its poor water solubility [194], being a class IV compound in the Biopharmaceuticals Classification System (BCS) (solubility: 0.052 mg/mL in water; lipophilicity: Papp = 0.037 × 10^−6^ cm/s) [195]. Despite its low solubility, the bioavailability of baicalein is still superior to that of baicalin when administered at equivalent doses in rats, when measuring the baicalin plasma concentration [196]. Many technological attempts have been made to improve the bioavailability of baicalein or baicalin: preparation of micelles with different excipients [194,197]; co-crystal formation with theophylline [198], nicotinamide [199], or other compounds [200]; co-precipitates with polyvinylpyrrolidone [201]; nanocrystals for oral or pulmonary administration [56,202,203,204]; nanoemulsions [205,206,207,208]; nanosuspensions [209]; nanoliposomes [210,211] and nanostructure lipid carriers [212,213]; self-assembled nanoparticles [214]; solid lipid nanoparticles [215]; solid dispersions with different excipients [216,217,218,219]; preparation of complexes with cyclodextrins [220]; and self-microemulsifying systems [221].

In rats, 75.7% of the dose of baicalein administered by i.v. injection was circulating under the form of conjugate (glucuronide, sulfate) metabolites. The absolute absorption of baicalein was estimated in rats to be about 40% [222]. In rats, at least, the baicalin concentration is higher following the oral administration of baicalein than following the administration of baicalin (the relative absorption of baicalin was only 65% of that of oral baicalein, and the absorption of the glucuronide was slower), therefore, it has been suggested that using baicalein should be preferred over using baicalin for pharmacological purposes [168,222]. Although the levels of baicalein were reported to be very low [223,224] or even undetected [100,225,226] following its oral administration in rats, the PK (in the same animal species) of a Japanese traditional medicine, Saiko-keishi-to, containing a complex mixture of herbal products, indicated a higher level of free baicalein and conjugated baicalein in plasma than the level of free and conjugated baicalin [227]. In a study of a Chinese mixture tablet (Yan-Ke-Ning), although the baicalein plasma level was inferior to that of baicalin, its AUC_0–t_ was far from being low, and was close to the level of baicalin (1201.50 ± 239.09 and 957.63 ± 187.08 ng·h /mL, respectively). In monkey, the absolute bioavailability of baicalein following administration of oral doses of 50–500 mg/kg varied between 13.1% and 23.0% [228].

It has been stated that wogonin has a very low oral bioavailability (1.10%), a consequence of its low solubility and extensive intestinal as well as hepatic first pass metabolism [74]. In one study, it was also reported that no free wogonin was detected in plasma following administration of an extract containing wogonin; instead, glucuronidated forms were dominant [226]. In another, with pure wogonin administered in rats by oral route (5 mg/kg), the plasma concentration of wogonoside was about 100 times higher than that of free wogonin [229].

It has been shown in rats that although in an extract of *Scutellariae radix* the amounts of baicalein were about four times those of oroxylin A, the systemic exposure of the two flavones was similar; likewise, although the amount of baicalein was comparable to that of wogonin, its systemic exposure was only around half of that of wogonin. The practical implication of such findings is that although oroxylin A derivatives in this herbal extract are lower quantitatively than baicalein derivatives, the clinical effects of the extract may be in fact equally influenced by the minor flavone derivatives [32].

Chrysin has also poor bioavailability and, similarly to baicalein, various approaches have been attempted in order to increase it, such as co-crystals with cytosine and thiamine hydrochloride [230], and nanoparticles [231].

Solubilized with hydroxypropyl-beta-cyclodextrin, after an oral dose of 50 mg/kg administered in rats, the synthetic flavone had an oral bioavailability of 8.54%, which is quite small, but better than that of wogonin (1.1%) [60]. Following pulmonary administration in rats, the bioavailability of GL-V9 was 93% higher than in oral administration [60].

## 12. Inter-Species Differences

The available non-clinical data indicate that there are considerable species differences in the behavior of the metabolizing enzymes against flavonoids in general and flavones in particular [232]. In vitro data derived by the same team of investigators from using a human jejunum S9 fraction and rat intestinal microsomes in equivalent experimental conditions indicated that the same conjugates form in the two species, but the rate of conjugate formation was generally smaller (in many cases substantially smaller) in rodents than in humans [160].

It is known that for a non-flavonoid compound (estragol), sulfation has been reported to be about 30 times more effective in male rat liver (data generated with S9 fraction) than in human liver (S9 data), while the efficiency was somewhat similar between human and mouse [233]. On the other hand, sulfation of 7-hydroxycoumarin was reported to have no noticeable difference between human, rat, or mouse species [234]. In the case of 7-hydroxy-flavone (7HF) and 6-hydroxy-flavone (6HF), the sulfation rate decreased from dog to rat, human, and mouse. In the case of 4′-hydroxy-flavone (which is a flavone substituted on the B-ring), the rates of sulfation among the four species are different: dog > rat > mouse > human [232]. Baicalein-6-*O*-sulfate is detected in human plasma, as shown by PK clinical trials, whereas in rats, the same conjugate is not formed to a substantial extent [45].

The elimination of baicalein varies considerably from species to species, the intrinsic clearance being remarkably smaller in humans than in other species. In decreasing order, the speed of oroxylin A formation from baicalein (baicalein methylation and, indirectly, the speed of elimination of baicaline from the organism, because methylation is the limiting step) decreases from rabbit to human in the following order: rabbit > pig > rat ≈ mouse > dog > guinea pig > monkey > human [29].

Such inter-species differences strongly indicate that it is important to exercise caution when extrapolating findings from non-clinical experiments to humans.

## 13. Intra-Species Differences (PK Variability)

The pharmacokinetics of several of these flavones has been shown to manifest considerable variations from one individual to another, and a number of variables have been identified that associate themselves with changes in the pharmacokinetics of these molecules. Human clinical data have indicated that chrysin PK shows ample interindividual variability, with C_max_ for the parent compound varying between 3 and 16 ng mL^−1^ and AUC between 5 and 193 ng mL^−1^ h [154]. In rat, the plasma concentrations of baicalin have also been reported to be highly variable [235], a finding also confirmed to a certain extent in human subjects; the t_max_ seemed to vary even more in humans than the C_max_ [191,236].

This high variability may be explained, at least to a good extent, by the involvement of gut microbiota in the metabolism and absorption of these flavones. In rats pre-treated with antibiotics for 3 days, the PK parameters of baicalin were substantially modified [237], and in experiments, similar findings were seen, e.g., the absolute bioavailability of baicalin decreased from 2.2 ± 0.2% in “normal” rats to 1.5 ± 0.2% in rats treated with antibiotics [30,238,239]. Since the microbiota may vary considerably from person to person and even in the same subject along time (although neither age nor gender could explain this variability in one study [240]), and is likely to be influenced by food and other exogenous and endogenous variables, it is reasonable to expect that the PK parameters of baicalin or similar flavones will be subject to fluctuations. This has been confirmed for both baicalin/baicalein and wogonoside/wogonin in a small human PK study [177]. Examination of the PK of baicalein and baicalin following the administration of a single 400 mg dose of baicalein in humans indicated that taking it with food increases the AUC of baicalein by 36% and decreases the AUC of baicalin by 22%, a difference assumed vaguely by the authors of the study to be related to the change in absorption, metabolism, or enterohepatic recycling of baicalein. Because baicalin is not an inactive metabolite, it is not possible to estimate the potential clinical impact of taking baicalein with or without food and to formulate a clear recommendation in this sense [241].

In vitro data (with their intrinsic limitations) have suggested that co-administration of baicalein, wogonin, and oroxylin A leads to increased absorption, as well as decreased metabolism of those flavonoids [242]. Significant differences have also been reported in the PK parameters of baicalein when administered in equal doses as a pure substance or as part of a purified extract of *S. baicalensis* (increase of the AUC in the latter case, attributed to increase absorption) [243]. Co-administration of *Scutellariae radix* extracts with different other herbal species has been associated with variations in the pharmacokinetics of its main active compounds (baicalin, wogonoside, oroxylin A-7-*O*-glucuronide) in both senses (increases or decreases in AUC, C_max_, or t_1/2_), explained by a variety of mechanisms: inhibition of beta-glucuronidase, competition among the active constituents of the herbs, increased metabolism or slower elimination, inhibition of MRP expression or function, inhibition of tight junction protein ZO-1 or actin, increased distribution, etc. [244].

Co-administration of a *Coptis chinensis* Franch. extract with a *Scutellariae radix* extract in rats has shown that the former, putatively through its berberine active ingredients, leads to a significant reduction of the main PK parameters (C_max_ and AUC), mediated by the inhibition of the intestinal microflora hydrolyzation of the flavonoid glycosides, as well as through their transportation across the intestinal mucosa [245]. The PK of baicalin, wogonoside, and oroxylin A glucuronide, showed obvious differences between administration as single herb (Huangqin-Tang) or as a compound prescription, with an increase in C_max_ and AUC_0—lim_, particularly for wogonoside [246]. In the PK analysis of a Chinese herbal mixture (Hu-gan-kan-kang-yuan capsule), the half-lives of wogonin and oroxylin A were much longer (around 40 h for both) than those reported in the literature, a difference probably also related to the influences of the other components of the formulation [247]. Co-administration of extracts from *Scutellariae herba* and *Acacia catechu* (L.f.) Willd. (peeled branches) indicated a significant decrease in C_max_ and AUC for baicalin, attributed by the authors to competitive inhibition of gastro-intestinal absorption [248]. Garlic co-administration seems also to have a negative impact on baicalin exposure in rats and on its therapeutic effects [249]. Puerarin increased considerably the absorption of baicalin in mice [250], whereas pre-treatment of rats with ursodeoxycholic acid led to a sizeable decrease in baicalin exposure, hypothesized to be related to an increase efflux of the flavone by MRP2 [251]. Co-administration of baicalin from a herbal product with levofloxacin injection in rats resulted in an increase in the AUC and half-life of baicalin [252], as did acupuncture applied to rats [253]. The PK parameters of wogonin from *Scutellariae radix* were reported to be decreasing when co-administered with rhubarb anthraquinones, through a decline in absorption [254]. Oxymatrine, by contrast, had no influence on the pharmacokinetics of baicalin [255]. The pharmacokinetic parameters of baicalein, wogonin, and oroxylin A also vary across other different herbal preparations [92,94,256,257,258,259], depending on their complex matrices of ingredients. It is likely that such reciprocal influences contribute to further variability of the PK of these flavones.

As shown above, wogonin has a very low bioavailability when administered alone; however, following the administration of a *Scutellaria barbata* extract, the free forms of wogonin represented about 35% of the total wogonin measured in rat plasma [260], which also indicates that the complex matrix of other ingredients from herbal products may exert a sizeable influence on its pharmacokinetics.

Administering baicalein with and without food in human healthy subjects revealed that the AUC and C_max_ of the aglycone slightly increased when taken with meals, whereas AUC and C_max_ for baicalin slightly decreased when administered with food [261]. The AUC of baicalein following baicalin administration seemed to be slightly lower when the glycoside was administered after a meal in comparison with the values obtained when administered before a meal [262], but the differences were small and is not clear whether they are of any clinical relevance.

The specific dosage form used to administer the flavonoids or the extraction process applied to the herbal product(s) containing the flavones may also exert an influence on the PK, as it has been shown that from a concentrated powder, the C_max_ and AUC of baicalein (sulfate/glucuronate) and wogonin (sulfate/glucuronate) are lower (in rats) than those of the herbal product administered as a traditional decoction [263]. In another study, it was reported that decoction (of Fuzi Xiexin Tang) ensured a higher content of baicalin, wogonoside, and wogonin in the extract than a macerate, but the bioavailabilities of these flavones in the macerate were several times higher than those of the same flavones from the extract obtained by decoction [264]. However, for another product (Sanhuang Xiexin Tang), the decoction ensured both better extraction and better bioavailability of flavones than maceration [265], indicating that the herbal matrix is important and such findings may not necessarily be extrapolated to other products. Wine processing of Chinese traditional medicines has also also associated with significant changes in the PK of chrysin, baicalein, wogonoside, oroxylin A-7-*O*-glucuronide, oroxylin, and baicalin, with an increase in the AUC_0–t_ [266] as well as differences in the tissue distribution of the flavones [267].

Pathological states or conditions may also have an impact on the pharmacokinetics, most evidence available for the moment being derived from baicalein or baicalin experiments. In rats, the reported t_max_ for baicalin (28 mg/kg, orally) was 3 h in normal animals, but significantly (*p* < 0.05) longer in febrile rats (5 h); similarly, t_1/2_ was shorter in normal rats (2.60 ± 1.42 vs. 5.68 ± 0.483), and the mean residence time was slightly inferior in the normal animals as compared with the febrile ones: 7.19 ± 1.36 h vs. 9.18 ± 0.513. Instead, C_max_ was reportedly higher (almost double) in the normal animals (860 ± 194 ng/mL vs. 411 ± 209 ng/mL) [268]. Qualitatively, the differences among normal and febrile rats were similar for the metabolite baicalein 6-*O*-glucopyranuronoside. T_max_ was longer for the metabolite than for the parent compound (4 h and 6 h, respectively), t_1/2_ was similar to that of the parent compound in the normal animals, but longer in the febrile rats (2.54 ± 1.31 vs. 4.68 ± 0.505). The mean residence time was similar to the parent compound for all animals, whereas the C_max_ was higher for the metabolite than for the parent compound (1397 ± 280 ng/mL in normal rats, 947 ± 353 in febrile rats) [268]. In another rat study, both C_max_ and AUC were higher in febrile rats than in normal ones, and the sick animals also had delayed absorption and elimination of the flavones from a Chinese traditional medicine [269].

The exposure of baicalin and also of wogonin and wogonoside was significantly higher in diabetic rats (models induced with streptozotocin) than in healthy rats, and this seemed to be related not to an increase of absorption (the intestinal permeability for baicalin rather decreased), but to an increase in the activity of β-glucuronidase [270,271,272,273,274,275]; a delay in gastric emptying and thus prolongation of the mean residence time in the gastro-intestinal tract of the flavones was also suggested as possibly contributing to this increase in exposure in diabetic animals [275]. A similar effect was also reported in diabetic mice (model of diabetes used in the experiment not reported), the sample of which also contained oroxylin A and baicalin (unlike the non-diabetic mice used as a control) [276]. In one study, oroxylin A was detected as a metabolite of baicalein in diabetic rats only, and not in otherwise healthy animals [277]; since baicalein was administered as part of a *Scutellariae herba* extract, it is not certain that the difference was directly related to the diabetic condition of the animals, but it seems a reasonable explanation that needs confirmation. Chrysin from a herbal extract (*Alpinia oxyphylla* fruit ethanol extract) considerably increased AUC and C_max_ in dementia rats when compared with normal rats [278]. The absorption and bioavailability of baicalin from herbal extracts was shown to be higher in rats with ulcerative colitis than in otherwise healthy rats [167,190]. In experimentally allergic rats, though, no differences were seen in the pharmacokinetics of baicalin when compared with normal controls [279].

Baicalin was reported to have a faster and better absorption as well as metabolism in rats with middle cerebral artery occlusion (MCAO), as compared with sham-operated rats [280]. Moreover, a liposome preparation intended to improve its bioavailability led to a higher distribution of the flavone in brain, whereas it could not be detected in the brain tissues of healthy control animals [281]. In the same rodent species, cerebral ischemia–reperfusion [282] and intrahepatic cholestasis [283] were associated with a significant increase in exposure and decrease in clearance of baicalin. It is not clear, though, to what extent these data are relevant and extrapolable to human clinical practice.

Wogonin was absorbed faster from a herbal extract in the diarrheal jejunum than in a normal one [284], and the plasma concentration was higher than the concentration measured in normal animals at almost all time points (between 0 and 40 h) [285]. There were also differences in the baicalin plasma concentrations among normal and diarrheal minipigs, but they were not consistent across time [285]. The exposure to wogonin increased by about 50% in rats when the flavonoid was co-administered with docetaxel (AUC to infinity 118.26 ± 30.14 vs. 78.37 ± 10.23) [192]. The AUC_last_ of baicalin and wogonin were 2.0- and 5.4-fold larger in a group of rats in which inflammation was induced with carrageenan (i.p.), as compared with the control group (with no inflammation) [286].

It has been speculated that because baicalin is transported by MRP2, which is known to have multiple single nucleotide polymorphisms, its disposition would vary among different individuals depending on their MRP2 mutations [47], although clinical evidence in support of this has not (yet) been published. In a MRP2-deficient rat strain, nonetheless, it has been shown that following administration of baicalein, the plasma concentration of its glucuronide derivative baicalin increases significantly, as a consequence of an important decrease in its biliary excretion and an increase of the sinusoidal efflux from the liver [47].

## 14. Linearity and PK Parameters

The volume of in vivo PK data is limited for this class of flavones, and data in humans are still more limited. Although baicalein has been reported in certain PK studies (e.g., [194]), other authors reporting such PK data have stated that “the content of baicalein cannot be detected after oral administration, the baicalin is predominant in the plasma when baicalein is administered orally, so baicalein absorption can be assessed by detecting the baicalin concentration and baicalein glycosides” [195].

For 5,7-DMF, it has been stated that following i.v. injection, there is linearity between dose and concentration in the interval of 10–50 mg/kg [120]. The dose proportionality of baicalein for C_max_ and AUC was inconclusive, particularly because of an anomaly observed for the 400 mg dose (more accurately, disproportionality between the 400 and 800 mg doses), assumed by the authors to be explained by enterohepatic recycling of the baicalein and saturation kinetics of the baicalein conversion to baicalin, with a possible contribution of intestinal microbiota [191]. The pharmacokinetics of baicalin was reported to be non-linear in humans [83], although in rat it was reported to be linear at 21.9 mg/kg (in a complex herbal matrix) [287]. For wogonin, both PK linearity [192] and non-linearity [74] have been reported (in rats), but even the data from the reference claiming linearity actually suggest a certain deviation from linearity.

A synthesis of the most important PK parameters of different B-ring unsubstituted flavones, in different species, doses and routes of administration is shown in Table S1 (Supplementary Data).

## 15. Knowledge Gaps and Directions of Future Research

The number of human clinical PK data on this subgroup of flavones is very limited, and data obtained in non-clinical experiments, which are more extensive, may in fact be deceiving. Some of the data on flavone metabolism were obtained with in vitro models of which the relevance for a human setting is impossible to assess (e.g., data obtained with Hep2G or HeLa cells, and even data obtained using Caco-2 cells). The dangers of extrapolating from limited non-clinical data may be illustrated by the initial research on chrysin metabolism, wherein, using microsomes from the Aroclor 1254-induced rat, it was shown that this flavone is mono- and dihydroxylated on the B-ring. However, the authors also performed experiments on the “normal” rat microsomes, where chrysin was barely metabolized (to the two main conjugates, glucuronide and sulfate) [153]. Even in vivo data obtained from other species are not easily extrapolable to the human setting.

An example that has already been mentioned is that of chrysin: published in vitro data indicates that chrysin is predominantly glucuronidated by UGT1A1 [150], in this kind of experiment glucuronidation of chrysin clearly had preeminence over sulfation [137] and the reader of such studies might remain under the impression that glucuronidation is an important pathway of chrysin metabolism in humans. Data generated using Caco-2 cells also indicate that chrysin is metabolized to a glucuronide derivative and a sulfate metabolite [288], data repeated thereafter by other authors [160], and in the baso-lateral compartment, a glucuronide metabolite was the dominant one for chrysin [137]. In vivo data obtained ex vivo in mouse [289] and in vivo by injection in rat [290] confirm glucuronide conjugation as slightly more extensive than sulfate conjugation for chrysin; nevertheless, in the case of an intra-gastric administration in rat, no parent compound and no sulfate conjugate was detected in plasma, only the glucuronide [290]. However, PK data published in a small human clinical trial (*n* = 7), have shown that in all cases, the glucuronide contribution to chrysin metabolism is trifling, and only the sulfate is prominent and relevant [154]. Nonetheless, this finding is consistent with those in vitro data showing that chrysin is a substrate of sulfotransferase, whereas flavonols are inhibitors of that enzyme [178]. One may be tempted to hypothesize that this could be a chance finding, or due to some unknown source of bias. It is interesting that the small sample of subjects was quite diverse (5 Caucasian, one Black, and one Asian; two females and five males), and in none was the glucuronide in amounts allowing quantification. It is therefore necessary to take into account the reliability of data depending on the nature of the experiment and species used: in vitro and non-clinical data have to be regarded with a higher dose of scientific skepticism than clinical data, the only that may truly be considered direct evidence. Moreover, the case for sulfation over glucuronidation in humans is strengthened by the data available for baicalein, where, unlike the rodent data, sulfation is also the predominant pathway of conjugation, not glucuronidation [177]. In the case of wogonin, though, the sulfates were roughly equivalent in amount to glucuronides, but even in this case they were not negligible, as the available non-clinical data would tend to suggest [177].

Although in vitro data have indicated that co-administration of mefenamic acid (which is metabolized by glucuronidation) with baicalein, wogonin, or oroxylin A would to lead to a significant increase in permeability and decrease in metabolism of the three flavonoids [291], an in vivo study (from the same research group) showed that the changes in AUC or C_max_ of the flavonoid by the mefenamic acid (and vice versa) were insignificant or inexistent [292]—another case where in vivo data disprove what the in vitro indicated at first.

Chrysin nanoemulsions formulated with sodium oleate were shown in rat pharmacokinetic studies to lead to a 4.3-fold increase in chrysin AUC and a 3.5-fold increase in C_max_, unlike nanoemulsions prepared with Tween 80. This was explained through inhibition of the UGT-catalyzed glucuronidation [293]. If the metabolism is different between humans and rodents, as the clinical data currently available seem to indicate (but derived from a sample of only seven subjects), with glucuronidation playing a substantially less important role in the case of humans, does such a nano-emulsion still remain relevant for increasing chrysin absorption in humans, where sulfate conjugation has been thought up to now to be the main metabolic pathway?

The metabolites of the different flavone molecules are known only to a little extent, and the potential biological effects of most of them are even less known. For instance, whereas for baicalin, the focus is usually on the parent compounds, baicalein and oroxylin A-7-*O*-β-D–glucuronide, 32 metabolites have been reported in rats using modern, sensitive tools such as UHPLC coupled with linear ion trap–Orbitrap mass spectrometry [170]. How many of these are pharmacologically relevant in vivo? The authors of the study assumed that ‘six of them’ might be the answer, but to what extent this assumption is true, further data will have to establish.

Limited data have suggested that methylated flavones are more stable than hydroxylated flavones, and 5,7-DMF was sizably more stable from a metabolism point of view than 3′,4′-DMF. This would suggest that B-ring unsubstituted flavones are metabolically more stable than those substituted on the B-ring, but may this fact also be extended to other molecules from the same two classes? Currently, we don’t know.

For baicalein and baicalin (which tend to inter-convert at various sites in the body) it has been demonstrated that absorption and total exposure are heavily influenced by the gut microbiome [32,140,294]. There seems to be no key reason for which the same would not be applied to other flavones, and an in vitro study of 13 flavonoids has shown that many flavonoids are actually more extensively degraded by intestinal microbiota than baicalein [140]. To what extent the different unsubstituted B-ring flavones are affected by intestinal microflora, and what the impact is on their pharmacokinetics, is currently little known and needs further investigation (it is likely to contribute to their enteric and enterohepatic recycling).

Moreover, because the glucuronide and sulfate conjugates have physicochemical properties that are quite distinct from those of the parent compounds, extrapolation of clinical effects from in vitro data performed with the parent compounds seems highly speculative and little justified [164]. Most of the glucuronides tend to be pharmacologically inactive, although notable exceptions are known [226,295]. Even when active, there may be differences favoring the aglycones: baicalin (glucuronide of baicalein) showed a weaker activity of promoting phagocytosis by macrophages than its aglycone, baicalein, even when this was administered at a lower concentration (10 vs. 3 μmol/L) [296].

That being said, the literature is dominated by studies on non-conjugated flavones. About a decade ago, appeals were made for an increased focus of research on the phase II conjugates (glucuronide and sulfate derivatives) [226,297], but they seem to have had little effect among the relevant research circles. The in vitro data generated with aglycones are not completely irrelevant, as they may prove useful to generate hypotheses about certain administration routes (e.g., topical, pulmonary, or i.v.) or delivery systems (such as those explored for baicalin), but for oral use they seem of a very limited use, if at all. It has been suggested that through the multiple recycling mechanisms, flavonoid exposure in the gut may be prolonged sufficiently in order to allow them to have more biological effects in the intestine segment than their humble bioavailability would suggest [16,295]. Even assuming that all the in vitro data are relevant for the in vivo context, increasing the body of research performed with the phase II conjugates of flavonoids makes sense, because they tend to be dominant and might more easily find practical applications. The road to understanding the pharmacokinetics of this group of flavones has been long and dusty, and the end is still far off.

## 16. Conclusions

B-ring unsubstituted flavones are absorbed after a deglycosylation step; therefore, the aglycone structure is much more important for their pharmacological effects than the glycoside residue(s). Aglycones have good permeability and are quickly absorbed, whereas glycosides have poor permeability and their oral absorption is slower. They tend to bind (to a variable extent) to serum albumin and are involved in several recycling circuits that prolong their residence time in the body. B-ring unsubstituted flavones tend to reach higher concentrations than plasma in liver and kidney, and lower concentrations in the pancreas or lung; the ability to penetrate the brain–blood barrier varies with the chemical structure, but it tends to be rather limited, if any. Most flavones of this group tend to be rapidly and intensely metabolized in the small intestine (where there is an important contribution of the local microbiota to their metabolism) and further in the liver, leading to low bioavalabilities. Glucuronidation and sulfation are the most important metabolic pathways when administered orally in the human body. Discrepancies between non-clinical and human clinical data indicate the need to cautiously interpret results generated in studies performed in vitro or in different animal species. Intra-species, the pharmacokinetics may vary considerably depending on the herbal matrix (i.e., what other compounds accompany a flavone molecule of interest) or particular pathological states.

## Figures and Tables

**Table 1:** Solubilities for most known unsubstituted B-ring flavones.

Substance	Water Solubility
5,7-dihydroxy-3-(3-methylbut-2-enyl)-flavone	14.61 mg/L
5,7-dihydroxy-3-[(2E)-5-methylhexa-2,4-dienyl]-flavone	7.10 mg/L
5,7-dimethoxyflavone	0.016 g/L
5-hydroxy-7-methoxy-3-methyl-flavone	53.67 mg/L
5-hydroxyflavone	80.61 mg/L
6,8-diprenyl-chrysin	2.40 mg/L
6-prenyl-chrysin	13.66 mg/L
7-hydroxyflavone	84.92 mg/L
Alnetin	28.80 mg/L
Baicalein	0.15 g/L
Baicalin	1.72 g/L
BA-j	15.92 mg/L
Chrysin (5,7-dihydroxyflavone)	0.1 g/L
Chrysin-5-methyl-ether	51.66 mg/L
GL-V9	18.25 mgL
Mosloflavone	40.04 mg/L
Moslosooflavone	37.09 mg/L
Negletein	66.94 mg/L
Norwogonin	0.16 g/L
Oroxylin A	66.60 mg/L
Oroxylin A-7-glucuronide	1.20 g/L
Pediflavone (isowogonin)	69.01 mg/L
Primetin	0.11 g/L
Tectochrysin	52.22 mg/L
Wogonin	65.34 mg/L
Wogonin-7-glucuronide	1.21 g/L

## Supplementary Materials

The following is available online at https://www.mdpi.com/1999-4923/11/8/370/s1, Table S1: Pharmacokinetic parameters of B-ring unsubstituted flavones in different species.
