# Supplementary material for: Pharmacokinetics of B-Ring Unsubstituted Flavones
Source: Pharmaceutics. 2019 Aug 1;11(8):370. doi: 10.3390/pharmaceutics11080370 (PMC6723510; doi:10.3390/pharmaceutics11080370)
Supplement: Supplementary file 1 [file pharmaceutics-11-00370-s001.pdf]

pharmaceutics

Supplementary Materials: Pharmacokinetics of B-Ring Unsubstituted Flavones

Robert Ancuceanu, Mihaela Dinu, Cristina Dinu-Pirvu, Valentina Anuţa and Vlad Negulescu

Table S1. Pharmacokinetic parameters of B-ring unsubstituted flavones in different species.

Compound Administered/ Measured	Species	Dose and route	Cmax (ng/ml)	AUC 0–t h (µg h/ml)	AUC 0–∞(µg h/ml)	t_{max} (h)	$t_{1/2}$ (h)	MRT 0–∞(h)	Reference
Baicalein/ Baicalein	Rat	75 mg/kg, oral	1610 ± 370	11.078 ± 1.46	12.01 ± 4.73	2.83 ± 1.10	-		[1]
Baicalein/ Baicalein	Rat	40 mg/kg, oral	1380 ± 1230	22.93 ± 13.13	40.99 ± 10.23	1.34 ± 0.45	3.45 ± 0.33	5.42 ± 1.04	[2]
Baicalein/ Baicalein	Rat	25 mg/kg (suspension), oral	1430 ± 170	7.96 ± 0.44	-	4.23 ± 0.26	3.75 ± 0.94	-	[3]
Baicalein/ Baicalein	Human	200 mg, single dose, oral, chewable table	13.69 ± 6.24	79.2 ± 26.8	88.0 ± 29.8	4 (0.5–5)	8.54 ± 5.93	-	[4]
Baicalein/ Baicalein	Human	400 mg, single dose, oral, chewable table	23.00 ± 11.76	122.2 ± 44.5	147.0 ± 44.9	1.25 (0.17–5)	18.85 ± 17.47	-	[4]
Baicalein/ Baicalein	Human	800 mg, single dose, oral, chewable table	28.65 ± 11.22	229.2 ± 60.6	278.3 ± 89.2	4 (0.33–5)	17.45 ± 9.84	-	[4]
Baicalein/ Baicalin	Human	200 mg, single dose, oral, chewable table	163.40 ± 109.73	1274.1 ± 900.0	1358.3 ± 918.8	4 (0.5–10)	9.69 ± 7.7	-	[4]
Baicalein/ Baicalin	Human	400 mg, single dose, oral, chewable table	274.69 ± 145.40	2488.0 ± 1080.8	2764.7 ± 1211.3	1 (0.17–5)	12.19 ± 11.56	-	[4]
Baicalein/ Baicalin	Human	800 mg, single dose, oral, chewable table	395.83 ± 199.06	3726.2 ± 2046.5	4563.9 ± 2910.6	3.5 (0.5–5)	16.35 ± 12.26	-	[4]

Compound Administered/ Measured	Species	Dose and route	Cmax (ng/ml)	AUC 0–t h (μg h/ml)	AUC 0–∞(μg h/ml)	t_{max} (h)	$t_{1/2}$ (h)	MRT 0–∞(h)	Reference
Baicalein/ Baicalein	Human	200 mg, multiple dose, oral, chewable table	17.610 ± 7.810	154.2 ± 81.0	169.0 ± 100.0	4 (1.5–5)	8.8 ± 4.85	-	[4]
Baicalein/ Baicalein	Human	400 mg, multiple dose, oral, chewable table	31.72 ± 13.41	296.9 ± 152.4	332.9 ± 180.0	4.5 (0.5–5)	12.53 ± 4.7	-	[4]
Baicalein/ Baicalein	Human	800 mg, multiple dose, oral, chewable table	65.17 ± 31.79	549.66 ± 252.2	598.4 ± 314.6	0.92 (0.17–4)	11.09 ± 6.27	-	[4]
Baicalein/ Baicalin	Human	200 mg, multiple dose, oral, chewable table	232.60 ± 130.25	2447.0 ± 2014.5	2799.2 ± 2656.6	1.5 (0.33–5)	9 ± 5.28	-	[4]
Baicalein/ Baicalin	Human	400 mg, multiple dose, oral, chewable table	823.83 ± 678.05	6020.5 ± 4197.5	7267.1 ± 5664.1	1.5 (0.33–5)	15.01 ± 9.21	-	[4]
Baicalein/ Baicalin	Human	800 mg, multiple dose, oral, chewable table	1076.23 ± 527.44	7053.0 ± 3886.7	8651.1 ± 7144.1	0.5 (0.33–2)	15.59 ± 16.42	-	[4]
Baicalein/Baicalein	Human	400 mg, fasted condition, oral	15.75 ± 9.33	89.47 ± 48.63	93.36 ± 50.11	3.5 (0.17–5)	5.39 ± 3.11	-	[5]
Baicalein/Baicalein	Human	400 mg, fed condition, oral	19.57 ± 9.44	122.07 ± 28.29	126.21 ± 28.27	4.5 (3–8)	5.74 ± 2.08	-	[5]
Baicalein/Baicalin	Human	400 mg, fasted condition, oral	182.47 ± 99.56	1193.23 ± 693.41	1266.43 ± 866.66	3 (0.33–6)	6.36 ± 5.85	-	[5]
Baicalein/Baicalin	Human	400 mg, fed condition, oral	154.78 ± 100.83	933.92 ± 282.32	940.88 ± 286.19	3 (2–8)	4.38 ± 1.14	-	[5]
Baicalin/Baicalin	Rat	500 mg/kg [1 ml/100 g, the solution contained 50.11 mg/mL), oral	9.840 ± 1.545* 10,700 ± 622.2*	201.566 ± 12.893	227.782 ± 21.443	0.375 ± 0.137* 8.000 ± 2.191*	16.056 ± 4.376	15.86 ± 1.17	[6]

Compound Administered/ Measured	Species	Dose and route	Cmax (ng/ml)	AUC 0–t h (μg h/ml)	AUC 0–∞(μg h/ml)	t_{max} (h)	$t_{1/2}$ (h)	MRT 0–∞(h)	Reference
Baicalein/Baicalein	Human	100 mg, single dose, oral	5.82 (3.33)	0.022 (0.011)	0.025 (0.011)	3.5 (0.5–5)	1.90 (1.17)	-	[7]
Baicalein/Baicalein	Human	200 mg, single dose, oral	11.54 (4.10)	0.007 (0.019)	0.075 (0.018)	3 (0.5–5)	8.03 (3.07)	-	[7]
Baicalein/Baicalein	Human	400 mg, single dose, oral	20.47 (11.81)	0.098 (0.059)	0.102 (0.061)	3 (0.17–5)	4.82 (2.95)	-	[7]
Baicalein/Baicalein	Human	800 mg, single dose, oral	35.38 (19.17)	0.211 (0.077)	0.218 (0.078)	1.5 (0.17–4)	8.23 (1.84)	-	[7]
Baicalein/Baicalein	Human	1200 mg, single dose, oral	51.62 (46.50)	0.308 (0.164)	0.323 (0.165)	2 (0.17–5)	10.16 (4.07)	-	[7]
Baicalein/Baicalein	Human	1600 mg, single dose, oral	43.06 (22.67)	0.239 (0.071)	0.255 (0.080)	1.5 (0.5–4)	8.71 (2.00)	-	[7]
Baicalein/Baicalein	Human	2200 mg, single dose, oral	131.36 (140.25)	0.478 (0.263)	0.497 (0.260)	1.25 (0.17–5)	7.29 (4.16)	-	[7]
Baicalein/Baicalein	Human	2800 mg, single dose, oral	108.17 (83.16)	0.541 (0.176)	0.678 (0.305)	0.75 (0.33–4)	15.01 (14.97)	-	[7]
Baicalein/Baicalin	Human	100 mg, single dose, oral	75.47 (26.43)	0.556 (0.137)	0.580 (0.136)	1.00 (0.5–4)	8.58 (5.06)	-	[7]
Baicalein/Baicalin	Human	200 mg, single dose, oral	182.00 (90.53)	0.980 (0.370)	0.983 (0.370)	3 (0.33–5)	4.22 (0.42)	-	[7]
Baicalein/Baicalin	Human	400 mg, single dose, oral	234.82 (140.48)	1.315 (0.761)	1.322 (0.767)	2.75 (0.33–5)	4.56 (1.71)	-	[7]
Baicalein/Baicalin	Human	800 mg, single dose, oral	428.58 (156.88)	3.853 (1.757)	3.878 (1.777)	1.5 (0.33–4)	4.84 (1.62)	-	[7]
Baicalein/Baicalin	Human	1200 mg, single dose, oral	770.00 (749.68)	4.888 (2.581)	5.177 (2.586)	0.5 (0.33–2)	10.80 (4.97)	-	[7]
Baicalein/Baicalin	Human	1600 mg, single dose, oral	635.85 (230.74)	3.839 (1.187)	3.918 (1.181)	1.5 (0.5–4)	8.64 (2.59)	-	[7]
Baicalein/Baicalin	Human	2200 mg, single dose, oral	2264.50 (3022.58)	7.392 (5.118)	7.500 (5.107)	0.75 (0.5–5)	5.87 (2.85)	-	[7]
Baicalein/Baicalin	Human	2800 mg, single dose, oral	1847.21 (1269.03)	10.349 (3.320)	11.962 (6.016)	1.25 (0.5–4)	9.65 (5.78)	-	[7]

Compound Administered/ Measured	Species	Dose and route	Cmax (ng/ml)	AUC 0–t h (μg h/ml)	AUC 0–∞(μg h/ml)	t_{max} (h)	$t_{1/2}$ (h)	MRT 0–∞(h)	Reference
Baicalein/Baicalin	Rat	210 mg/kg, oral	1530 ± 220	17.77 ± 0.66	-	0.087 ± 0.02	7.15 ± 1.58	9.46 ± 0.51	[8]
Baicalein/Wogonoside	Rat	210 mg/kg, oral	1230 ± 120	14.67 ± 1.93	-	0.11 ± 0.1	8.20 ± 0.42	9.74 ± 0.08	[8]
Baicalein/Baicalein	Monkey (*Rhesus*)	50 mg/kg, oral	167.3 ± 73.2	0.454 ± 0.1212	-	1.6 ± 0.3	1.4 ± 1.0	2.9 ± 0.3	[9]
Baicalein/Baicalin	Monkey (*Rhesus*)	50 mg/kg, oral	4216.7 ± 1380.7	18.366 ± 4.796	-	3.8 ± 0.5	4.1 ± 0.8	5.1 ± 0.4	[9]
Baicalein/Baicalein	Monkey (*Rhesus*)	150 mg/kg, oral	318.4 ± 51.8 1	1.156.1 ± 0.205	-	1.4 ± 0.6	6.4 ± 3.6	5.9 ± 0.8	[9]
Baicalein/Baicalin	Monkey (*Rhesus*)	150 mg/kg, oral	7194.7 ± 4984.5	47.655 ± 15.208	-	4.0 ± 0.8	4.1 ± 0.5	7.2 ± 2.5	[9]
Baicalein/Baicalein	Monkey (*Rhesus*)	500 mg/kg, oral	612.5 ± 316.0	2.527 ± 1.537	-	2.3 ± 1.2	13.4 ± 9.5	6.2 ± 1.5	[9]
Baicalein/Baicalin	Monkey (*Rhesus*)	500 mg/kg, oral	13687.0 ± 2215.8	104.717 ± 52.674	-	3.5 ± 0.6	6.5 ± 3.6	9.0 ± 4.1	[9]
Baicalein/Baicalein	Monkey (*Rhesus*)	10 mg/kg, i.v.	30409.2 ± 4610.7	7.421 ± 1.080	-	0.033	4.8 ± 5.0	0.4 ± 0.2	[9]
Baicalein/Baicalin	Monkey (*Rhesus*)	10 mg/kg, i.v.	6146.6 ± 3363.4	4.390 ± 0.860	-	0.083	0.9 ± 0.2	3.3 ± 0.4	[9]
Baicalein/Baicalin	Rat	121 mg/kg, oral	7180 ± 1250	71.41 ± 4.38	-	1.67 ± 0.29	-	-	[10]
Baicalein/Baicalein	Rat	121 mg/kg, oral	1240 ± 780	0.79 ± 0.08	-	0.167 ± 0	-	-	[10]
Baicalein/Baicalin	Rat	10 mg/kg, i.v.	16660 ± 540	14.91 ± 0.55	-	0.083 ± 0	-	-	[10]
Baicalein/Baicalein	Rat	10 mg/kg, i.v.	10430 ± 1160	4.03 ± 0.85	-	0.083 ± 0	-	-	[10]
Baicalin/Baicalin	Rat	200 mg/kg, oral	3390 ± .720* 3860 ± 570*	48.48 ± 7.94	-	0.20 ± 0.07* 8.40 ± 0.89*	-	-	[11]
Baicalin/Baicalin	Rat	12 mg/kg, i.v.	-	18.02 ± 3.45	-	-	5.70 ± 0.76	1.92 ± 0.47	[11]
Baicalin/Baicalin	Rat	224 μmoL/kg (100 mg/kg), oral	446.36 ± 53.56	1.98 ± 0.18	-	5.0	-	-	[12]

Compound Administered/ Measured	Species	Dose and route	Cmax (ng/ml)	AUC 0–t h (µg h/ml)	AUC 0–∞(µg h/ml)	t_{max} (h)	$t_{1/2}$ (h)	MRT 0–∞(h)	Reference
Baicalin/Baicalin	Rat	37 µmoL/kg (16.5 mg/kg), i.v.	-	14.98	-	-	0.12 ± 0.02	-	[12]
Baicalin/Baicalein conjugates	Rat	224 µmoL/kg (100 mg/kg), oral	783.7 ± 351.3	8.75 ± 3.65	-	6.59 ± 7.31	-	11.62 ± 5.35	[13]
Baicalein/Baicalein conjugates	Rat	224 µmoL/kg (60.5 mg/kg), oral	3675.3 ± 2432.16	13.42 ± 7.89	-	0.17 ± 0	-	10.86 ± 4.38	[13]
Baicalein/Baicalein	Rat	40 mg/kg, oral	2870 ± 1820	24.93 ± 13.13	40.99 ± 13.35	1.44 ± 1.05	2.32 ± 0.33	7.43 ± 1.04	[14]
Baicalein/Baicalein	Rat	80 mg/kg, oral	36.63 ± 7.59	0.20 ± 0.08	-	2.82 ± 1.76	10.98 ± 5.70	-	[15]
Baicalein/Baicalin	Rat	80 mg/kg, oral	2497.66 ± 483.30	23.02 ± 8.54	-	5.40 ± 3.13	1.77 ± 0.35	-	[15]
Baicalein/Baicalein	Rat	15 mg/kg, oral	45.8 ± 20.4	0.21 ± 0.04	-	0.08 ± 0.0	7.6 ± 3.2	11.6 ± 3.4	[16]
Baicalin/Baicalin	Rat	28 mg/kg, oral	860 ± 194	6.413 ± 2.711	6.439 ± 2.733	3	2.60 ± 1.42	7.19 ± 1.36	[17]
Baicalin/ baicalein 6-O-glucopyranuronoside	Rat	28 mg/kg, oral	1397 ± 280	11.529 ± 1.459	11.573 ± 1.867	4	2.54 ± 1.31	7.41 ± 1.33	[17]
Wogonin/Wogonin	Rat	10 mg/kg, oral	15.00 ± 4.21	0.033 ± 0.005	0.034 ± 0.006	0.54 ± 0.38	1.83 ± 1.12	-	[18]
Wogonin/Wogonin	Rat	20 mg/kg, oral	35.36 ± 3.64	0.070 ± 0.007	0.078 ± 0.0 10	0.38 ± 0.14	1.53 ± 1.43	-	[18]
Wogonin/Wogonin	Rat	40 mg/kg, oral	76.84 ± 12.69	0.219 ± 0.030	0.256 ± 0.042	0.58 ± 0.20	2.04 ± 2.76	-	[18]
Wogonin/Wogonin	Beagle dog	5 mg/kg, i.v.	6838.7 ± 1322.1	0.630 ± 0.112	0.632 ± 0.113	-	5.9 ± 6.9	0.12 ± 0.03	[19]
Wogonin/Wogonin	Beagle dog	native drug, 15 mg/kg, i.g.	2.5 ± 1.1	0.007 ± 0.002	0.011 ± 0.005	0.7 ± 0.3	10.1 ± 8.9	3.6 ± 0.2	[19]
Wogonin/Wogonin	Beagle dog	solid dispersion, 5 mg/kg, i.g.	7.9 ± 3.3	0.021 ± 0.003	0.022 ± 0.005	0.3 ± 0.2	5.1 ± 2.6	5.3 ± 3.0	[19]
Wogonin/(Free) Wogonin	Beagle dog	5 mg/kg, i.g.	12.3 ± 3.3	0.016 ± 0.006	0.018 ± 0.007	0.35 ± 0.21	4.94 ± 2.53	3.39 ± 1.25	[19]

Compound Administered/ Measured	Species	Dose and route	Cmax (ng/ml)	AUC 0–t h (μg h/ml)	AUC 0–∞(μg h/ml)	t_{max} (h)	$t_{1/2}$ (h)	MRT 0–∞(h)	Reference
Wogonin/Total wogonin after enzymolysis	Beagle dog	5 mg/kg, i.g.	156.5 ± 40.9	0.449 ± 0.165	0.555 ± 0.371	0.51 ± 0.13	9.1 ± 5.2	8.38 ± 3.14	[19]
Wogonin/Wogonin	Rat	10 mg/kg, i.v.	7120 ± 1220	1.852 ± 0.328	1.869 ± 0.315	-	0.236 ± 0.070	0.14 ± 0.10	[20]
Wogonin/Wogonin	Rat	20 mg/kg, i.v.	19130 ± 3060	4.771 ± 0.920	4.779 ± 0.917	-	0.243 ± 0.088	0.107 ± 0.049	[20]
Wogonin/Wogonin	Rat	40 mg/kg, i.v.	43800 ± 6140	12.607 ± 0.440	12.637 ± 0.449	-	0.224 ± 0.059	0.109 ± 0.012	[20]
Wogonin/Wogonin	Rat	100 mg/kg, i.g.	300 ± 80	0.264 ± 0.034	0.284 ± 0.035	-	0.466 ± 0.079	-	[20]
Wogonin/Wogonin	Rat	5 mg/kg, i.v.	-	0.874	-	-	0.38	0.35	[21]
Wogonin/Wogonin	Beagle dog	20 mg/kg, i.v.	-	-	2.138 ± 0.231	-	1.51 ± 0.43	0.14 ± 0.03	[22]
Oroxylin A/Oroxylin A	Rat	20 mg/kg, i.v.	-	0.343	0.344	-	4.8	-	[23]
Oroxylin A/Oroxylin A-7-O-glucuronide	Rat	20 mg/kg, i.v.	-	6.341	6.342	-	2.47	-	[23]
Chrysin/Chrysin	Human	400 mg, oral	-	64 (33)	-	-	-	-	[24]
Chrysin/Chrysin sulfate	Human	400 mg, oral	-	1490 (485)	-	-	-	-	[24]
Chrysin/Chrysin	Rat	2 mg/kg, iv	-	-	0.2759 ± 0.050	-	0.04 ± 0.01	-	[25]
Chrysin/Chrysin sulfate	Rat	2 mg/kg, iv	1,367.0 ± 468.8	-	0.418 ± 0. 190	0.10 ± 0.07	0.2 ± 0.1	-	[25]
Chrysin/Chrysin glucuronide	Rat	2 mg/kg, iv	2,310.2 ± 1,069.2	-	0.473 ± 0.178	0.03 ± 0.05	0.4 ± 0.6	-	[25]
Chrysin/Chrysin glucuronide	Rat	100 mg/kg, i.g.	364.6 ± 118.7		2.701 ± 0.963	3.6 ± 0.6	3.0 ± 1.9	-	[25]
Chrysin/Chryisin	Mouse	20 mg/kg, oral	2.542 ± 2.542	0.015 ± 0.007	-	4.00 ± 1.36	-	-	[26]
Chrysin/Chryisin-7-O-glucuronide	Mouse	20 mg/kg, oral	68.85 ± 17.21	0.860 ± 0.353	-	7.00 ± 1.15	-	-	[26]

Compound Administered/ Measured	Species	Dose and route	Cmax (ng/ml)	AUC 0–t h (µg h/ml)	AUC 0–∞(µg h/ml)	t_{max} (h)	$t_{1/2}$ (h)	MRT 0–∞(h)	Reference
Chrysin/Chryisin-7-O-sulfate	Mouse	20 mg/kg, oral	43.46 ± 13.37	0.441 ± 0.14	-	6.50 ± 1.00	-	-	[26]
GL-V9/GL-V9	Rat	50 mg/kg, oral	167.33 ± 50.05	1.212 ± 0.246	1.256 ± 0.272	0.31-0.77	3.08 ± 1.05	7.70 ± 0.91	[27]
GL-V9/GL-V9	Rat	5 mg/kg, pulmonary	1095.33 ± 151.24	1.319 ± 0.320	1.382 ± 371	0.16-0.40	3.44 ± 0.34	3.07 ± 0.66	[27]
GL-V9/GL-V9	Rat	5 mg/kg, i.v.	1.055 ± 0.186	1.419 ± 0.473	1.496 ± 0.468	0.03-0.08	4.11 ± 1.66	4.02 ± 1.14	[27]

* Values for bimodal PK reported.

References

1. Zhou, Y.; Dong, W.; Ye, J.; Hao, H.; Zhou, J.; Wang, R.; Liu, Y. A novel matrix dispersion based on phospholipid complex for improving oral bioavailability of baicalein: Preparation, in vitro and in vivo evaluations. *Drug Deliv.* **2017**, *24*, 720–728.
2. Shen, H.; Liu, Y.; Zhang, H.; Ding, P.; Zhang, L.; Zhang, L.; Ju, J. Enhancing the oral bioavailability of baicalein via Solutol® HS15 and Poloxamer 188 mixed micelles system. *J. Pharm. Pharmacol.* **2018**.
3. Yin, J.; Xiang, C.; Wang, P.; Yin, Y.; Hou, Y. Biocompatible nanoemulsions based on hemp oil and less surfactants for oral delivery of baicalein with enhanced bioavailability. *Int. J. Nanomedicine* **2017**, *12*, 2923–2931.
4. Pang, H.; Xue, W.; Shi, A.; Li, M.; Li, Y.; Cao, G.; Yan, B.; Dong, F.; Xiao, W.; He, G.; et al. Multiple-Ascending-Dose Pharmacokinetics and Safety Evaluation of Baicalein Chewable Tablets in Healthy Chinese Volunteers. *Clin. Drug Investig.* **2016**, *36*, 713–724.
5. Pang, H.; Shi, A.; Li, M.; Xue, W.; Li, Y.; Cao, G.; Yan, B.; Dong, F.; Xiao, W.; He, G.; et al. Simultaneous Determination of Baicalein and Baicalin in Human Plasma by High Performance Liquid Chromatograph-Tandem Spectrometry and its Application in a Food-Effect Pharmacokinetic Study. *Drug Res.* **2016**, *66*, 394–401.
6. Chen, H.; Li, Z.; Li, Y.; Wu, X.; Wang, S.; Chen, K.; Zheng, X.; Du, Q.; Tang, D. Simultaneous determination of baicalin, oroxylin A-7-O-glucuronide and wogonoside in rat plasma by UPLC-DAD and its application in pharmacokinetics of pure baicalin, Radix Scutellariae and Yinhuang granule. *Biomed. Chromatogr. BMC* **2015**, *29*, 1819–1825.
7. Li, M.; Shi, A.; Pang, H.; Xue, W.; Li, Y.; Cao, G.; Yan, B.; Dong, F.; Li, K.; Xiao, W.; et al. Safety, tolerability, and pharmacokinetics of a single ascending dose of baicalein chewable tablets in healthy subjects. *J. Ethnopharmacol.* **2014**, *156*, 210–215.
8. Zhang, Z.-Q.; Liua, W.; Zhuang, L.; Wang, J.; Zhang, S. Comparative pharmacokinetics of baicalin, wogonoside, baicalein and wogonin in plasma after oral administration of pure baicalin, radix scutellariae and scutellariae-paeoniae couple extracts in normal and ulcerative colitis rats. *Iran. J. Pharm. Res. IJPR* **2013**, *12*, 399–409.
9. Tian, S.; He, G.; Song, J.; Wang, S.; Xin, W.; Zhang, D.; Du, G. Pharmacokinetic study of baicalein after oral administration in monkeys. *Fitoterapia* **2012**, *83*, 532–540.
10. Zhang, J.; Lv, H.; Jiang, K.; Gao, Y. Enhanced bioavailability after oral and pulmonary administration of baicalein nanocrystal. *Int. J. Pharm.* **2011**, *420*, 180–188.
11. Liu, L.; Deng, Y.-X.; Liang, Y.; Pang, X.-Y.; Liu, X.-D.; Liu, Y.-W.; Yang, J.-S.; Xie, L.; Wang, G.-J. Increased oral AUC of baicalin in streptozotocin-induced diabetic rats due to the increased activity of intestinal beta-glucuronidase. *Planta Med.* **2010**, *76*, 70–75.

12. Xing, J.; Chen, X.; Zhong, D. Absorption and enterohepatic circulation of baicalin in rats. *Life Sci.* **2005**, *78*, 140–146.
13. Lai, M.-Y.; Hsiu, S.-L.; Tsai, S.-Y.; Hou, Y.-C.; Chao, P.-D.L. Comparison of metabolic pharmacokinetics of baicalin and baicalein in rats. *J. Pharm. Pharmacol.* **2003**, *55*, 205–209.
14. Fan, J.; Dai, Y.; Shen, H.; Ju, J.; Zhao, Z. Application of Soluplus to Improve the Flowability and Dissolution of Baicalein Phospholipid Complex. *Mol. Basel Switz.* **2017**, *22*.
15. Li, W.; Pi, J.; Zhang, Y.; Ma, X.; Zhang, B.; Wang, S.; Qi, D.; Li, N.; Guo, P.; Liu, Z. A strategy to improve the oral availability of baicalein: The baicalein-theophylline cocrystal. *Fitoterapia* **2018**, *129*, 85–93.
16. Yu, H.; Chang, J.-S.; Kim, S.Y.; Kim, Y.G.; Choi, H.-K. Enhancement of solubility and dissolution rate of baicalein, wogonin and oroxylin A extracted from Radix scutellariae. *Int. J. Pharm.* **2017**, *528*, 602–610.
17. Huo, X.-K.; Wang, B.; Zheng, L.; Cong, H.-J.; Xiang, T.; Wang, S.-M.; Sun, C.-P.; Wang, C.; Zhang, L.; Deng, S.; et al. Comparative pharmacokinetic study of baicalin and its metabolites after oral administration of baicalin and Chaiqin Qingning capsule in normal and febrile rats. *J. Chromatogr. B Analyt. Technol. Biomed. Life. Sci.* **2017**, *1059*, 14–20.
18. Wang, T.; Long, F.; Jiang, G.; Cai, H.; Jiang, Q.; Cheng, K.; Hu, Z.; Wang, Y. Pharmacokinetic properties of wogonin and its herb-drug interactions with docetaxel in rats with mammary tumors. *Biomed. Chromatogr. BMC* **2018**, e4264.
19. Zhu, N.; Li, J.-C.; Zhu, J.-X.; Wang, X.; Zhang, J. Characterization and Bioavailability of Wogonin by Different Administration Routes in Beagles. *Med. Sci. Monit. Int. Med. J. Exp. Clin. Res.* **2016**, *22*, 3737–3745.
20. Talbi, A.; Zhao, D.; Liu, Q.; Li, J.; Fan, A.; Yang, W.; Han, X.; Chen, X. Pharmacokinetics, tissue distribution, excretion and plasma protein binding studies of wogonin in rats. *Mol. Basel Switz.* **2014**, *19*, 5538–5549.
21. Tsai, T.H.; Chou, C.J.; Tsai, T.R.; Chen, C.F. Determination of wogonin in rat plasma by liquid chromatography and its pharmacokinetic application. *Planta Med.* **1996**, *62*, 263–266.
22. Peng, J.; Qi, Q.; You, Q.; Hu, R.; Liu, W.; Feng, F.; Wang, G.; Guo, Q. Subchronic toxicity and plasma pharmacokinetic studies on wogonin, a natural flavonoid, in Beagle dogs. *J. Ethnopharmacol.* **2009**, *124*, 257–262.
23. Liu, W.; Xu, X.; Feng, F.; Wu, C. Simultaneous Quantification of Oroxylin A and Its Metabolite Oroxylin A-7-O-Glucuronide: Application to a Pharmacokinetic Study in Rat. *Chromatographia* **2011**, *74*, 75–81.
24. Walle, T.; Otake, Y.; Brubaker, J.A.; Walle, U.K.; Halushka, P.V. Disposition and metabolism of the flavonoid chrysin in normal volunteers. *Br. J. Clin. Pharmacol.* **2001**, *51*, 143–146.
25. Noh, K.; Oh, D.G.; Nepal, M.R.; Jeong, K.S.; Choi, Y.; Kang, M.J.; Kang, W.; Jeong, H.G.; Jeong, T.C. Pharmacokinetic Interaction of Chrysin with Caffeine in Rats. *Biomol. Ther.* **2016**, *24*, 446–452.
26. Ge, S.; Gao, S.; Yin, T.; Hu, M. Determination of pharmacokinetics of chrysin and its conjugates in wild-type FVB and Bcrp1 knockout mice using a validated LC-MS/MS method. *J. Agric. Food Chem.* **2015**, *63*, 2902–2910.
27. Xing, H.; Ren, C.; Kong, Y.; Ni, Q.; Wang, Z.; Zhao, D.; Li, N.; Chen, X.; Lu, Y. Determination of GL-V9, a derivative of wogonin, in rat plasma by UPLC-MS/MS and its application to a pharmacokinetic study after oral and pulmonary administration. *Biomed. Chromatogr.* **2019**, e4556.
